# Biomineralization of bone tissue: calcium phosphate-based inorganics in collagen fibrillar organic matrices

**DOI:** 10.1186/s40824-022-00288-0

**Published:** 2022-09-06

**Authors:** Min-Ho Hong, Jung Heon Lee, Hyun Suk Jung, Heungsoo Shin, Hyunjung Shin

**Affiliations:** 1 Department of Dental Biomaterials and Research Institute of Oral Science, College of Dentistry, Gangneung-Wonju National University, Gangneung, 25457 Republic of Korea; 2 School of Advanced Materials Science and Engineering, Sungkyunkwan University, Suwon, 16419 Republic of Korea; 3 SKKU Institute of Energy Science and Technology (SIEST), Sungkyunkwan University, Suwon, 16419 Republic of Korea; 4 Department of Bioengineering, Hanyang University, Seoul, 04763 Republic of Korea; 5 BK21 Plus Future Biopharmaceutical Human Resources Training and Research Team, Hanyang University, Seoul, 04763 Republic of Korea; 6 Institute of Nano Science & Technology (INST), Hanyang University, Seoul, 04763 Republic of Korea; 7 Department of Energy Science, Nature Inspired Materials Processing Research Center, Sungkyunkwan University, Suwon, 16419 Republic of Korea

**Keywords:** Biomineralization, Hierarchical structure, Bone growth, Bone regeneration, Nucleation and crystallization, Collagen matrix

## Abstract

**Background:**

Bone regeneration research is currently ongoing in the scientific community. Materials approved for clinical use, and applied to patients, have been developed and produced. However, rather than directly affecting bone regeneration, these materials support bone induction, which regenerates bone. Therefore, the research community is still researching bone tissue regeneration. In the papers published so far, it is hard to find an improvement in the theory of bone regeneration. This review discusses the relationship between the existing theories on hard tissue growth and regeneration and the biomaterials developed so far for this purpose and future research directions.

**Mainbody:**

Highly complex nucleation and crystallization in hard tissue involves the coordinated action of ions and/or molecules that can produce different organic and inorganic composite biomaterials. In addition, the healing of bone defects is also affected by the dynamic conditions of ions and nutrients in the bone regeneration process. Inorganics in the human body, especially calcium- and/or phosphorus-based materials, play an important role in hard tissues. Inorganic crystal growth is important for treating or remodeling the bone matrix. Biomaterials used in bone tissue regeneration require expertise in various fields of the scientific community. Chemical knowledge is indispensable for interpreting the relationship between biological factors and their formation. In addition, sources of energy for the nucleation and crystallization processes of such chemical bonds and minerals that make up the bone tissue must be considered. However, the exact mechanism for this process has not yet been elucidated. Therefore, a convergence of broader scientific fields such as chemistry, materials, and biology is urgently needed to induce a distinct bone tissue regeneration mechanism.

**Conclusion:**

This review provides an overview of calcium- and/or phosphorus-based inorganic properties and processes combined with organics that can be regarded as matrices of these minerals, namely collagen molecules and collagen fibrils. Furthermore, we discuss how this strategy can be applied to future bone tissue regenerative medicine in combination with other academic perspectives.

## Background

Several studies on bone tissue regeneration are ongoing in different disciplines. Recently, an approach to problem-solving, that cuts across many different disciplinary boundaries, have attracted attention [1]. It combines knowledge, tools, and even mindsets derived from life, medical, physical, chemical, and engineering sciences. This can lead to a comprehensive framework for the scientific and social challenges at the interfaces across biological and chemical/physical sciences [2]. The clinical environment welcomes the rapidly growing bone regeneration technology.

From the perspective of bone tissue research, scholars working in different fields with a single target have different approaches to experimentation and analysis. The control of biological and material functions of hard tissues such as bones and teeth, through a combination of cells, materials, and biological factors has been considered a promising approach. These technologies regenerate and repair hard tissues. Although there are numerous research papers and reviews of these studies, there are only few examples of scientific interpretations that include both clinical translation and practical application. Their practical application in the field is not easy [3]. For this reason, it is difficult to conclude form the experimental results obtained through the analysis of different fields. For example, in the study of mineralization, some scientists have analyzed the crystal structure at each stage of nucleation, the physical and chemical environment of the human body, and the materials required for the mineralization process.

On the other hand, scientists in other fields want to explain how osteoblasts, osteoclasts, and other peripheral biological factors affect bone tissue, which directly affects bone growth and regeneration. This analytical process and conclusions lead to jargon, which is difficult to understand in materials science. This eventually leads to one goal: studying of bone growth and regeneration mechanisms, in which experts in various fields are keenly aware of the need for several collaborative studies and an objective theorizing process to improve our understanding of bone tissue. Heterogeneity can lead to conflicts due to differences in approaches to research problems, and diversity contributes to the competence to invent creative solutions that can go beyond the common paradigm [2].

**Fig. 1:**
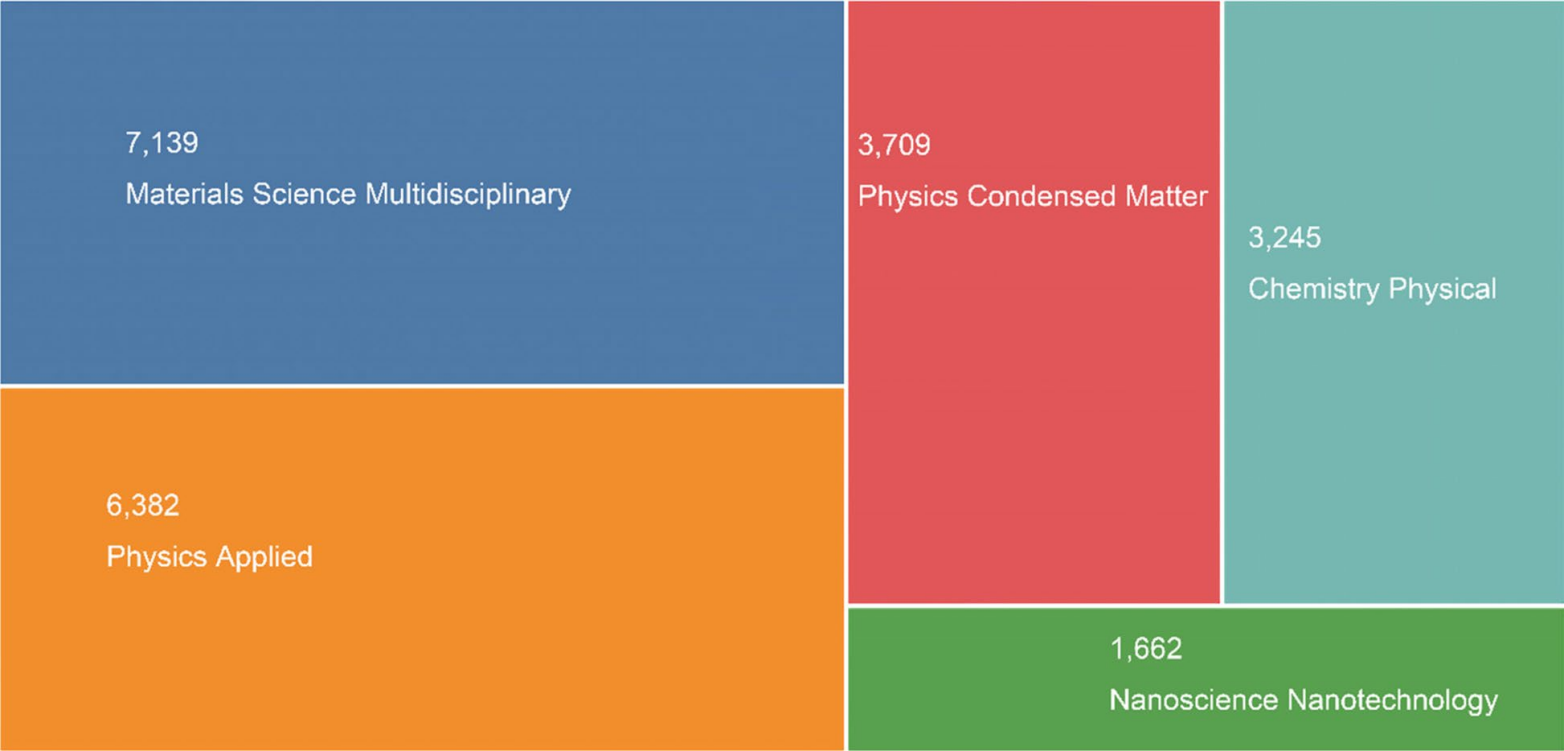
TreeMap Chart of publications for 15,943 results from Web of Science Core Collection between 2017 and 2021 (Data last updated: 2022–03-01)

The explosive interest in bone tissue regeneration using synthetic and/or fabricated biomaterials has recently been shown in part by research papers and citations in a rapidly growing field (Fig. 1). The results of the Web of Science search showed that articles on this topic have been steadily published in the last five years (15,943) (keywords: bone*tooth (theme) or materials or mineralization).

This review seeks to address these questions from a material science perspective. Biomineralization in the human body is based on tissue regeneration when a disease occurs, how to replicate the properties of mineralized tissue, and what technologies have been developed to identify such repair processes. This is explained based on the physical and chemical analysis techniques used to review the selected biotechnology approaches. First, the structure of bone tissue from a medical/biological point of view will be briefly explained, along with the main components of bone tissue, minerals, and collagen that serve as a substrate or matrix for mineral gorwth. Before discussing calcium-based materials, and minerals that make up bone tissue, various synthetic methods for developing synthetic bone graft materials will be briefly introduced. In the mineralization process, we discuss the latest research on the crystallization process by particle attachment after nucleation in materials and briefly explain the theory derived from the convergence of materials science and biology. Based on the knowledge and techniques described above, we explain the recent research on the biomineralization of bone tissue with methods for biomimetic synthesis in an environment like the actual human body that has been recently focused on. The results of the latest structural analytical techniques for bone tissue growth and regeneration are also reviewed, and the conclusion section describes the research direction that should be concentrated in the future.

### Bone structure and need for regeneration

The skeletal tissue that exists in the body is composed of inorganic [predominantly hydroxyapatite (HAp; Ca_10_(PO_4_)_6_(OH)_2_) crystals] and organic (osteoid) matter. Type I collagen (Col-I) is a protein that consists mainly of organic matter (80%) in bone tissue. Therefore, many researchers have attempted to synthesize self-manipulating Col-I for bone regeneration and regeneration of other tissues associated with Col-I [4–6]. However, no case has yet been synthesized identical to actual Col-I. Generally, collagen consists of twined amino acids that form triple-helices of elongated fibrils. The process for the growth of Col-I is as follows: initial collagen chains undergo several post-translational modifications, including proline and lysine residue hydroxylation, as well as glucose and galactose bonds to the hydroxylysine residues [7]. Transient binding between the molecular protector and the procollagen chain domain promotes the winding of a triple helix from the carboxy terminus to the amino terminus. After secretion, non-collagenous pro-peptides are removed by procollagen amino and carboxy proteinases. The processed collagen molecules bind in parallel but a staggered fashion, generating a fibril with a banding pattern that can be generally observed with an electron microscope. Although this is well known in theory, the scientific reality is that Col-I synthesized by scientists cannot have the same properties as natural Col-I owing to minute structural differences [8]. In addition, the synthesis process is time-consuming and economically inefficient, and researchers usually choose extraction from animals over Col-I artificial synthesis [9]. Table 1 summarizes the advantages and disadvantages of different collagen preparation methods [10].

**Table 1:** Distinct advantages and disadvantages of different collagen preparation methods [10], © 2018 WILEY‐VCH Verlag GmbH & Co. KGaA, Weinheim

Sources	Advantages	Disadvantages
Tissue extracted collagen	High yield No antigenic p-determinant	Potential of disease transmission
Cell synthesized collagen	Can be autologous	Low yield
Recombinantly produced collagen	Low immune response	Low yield Stability issues
Peptide synthesis produced collagen	Exclusion of allogeneic/xenogeneic issues	Low yield Assembly/registration issues

The inorganic component of bone tissue is generally known to be HAp; however, its structure and properties are now well understood, and various studies are still ongoing [11]. In addition to calcium and phosphate, minerals that can be called the minerals of bone contain significant amounts of carbonate, magnesium, sodium, and fluorine. Because of the chemical properties of each element, minerals of different elemental compositions exist as they are exchanged [12].

**Fig. 2:**
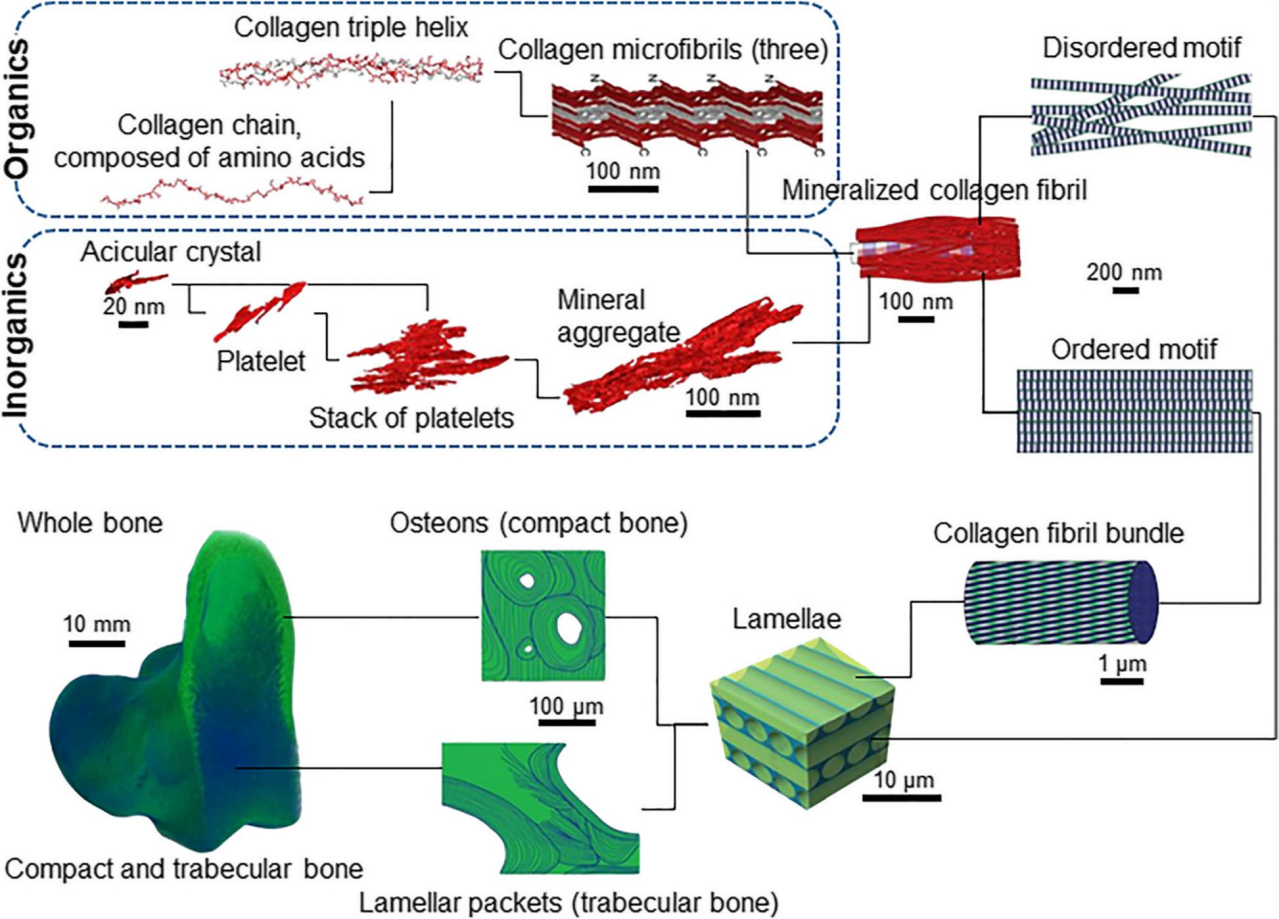
A scheme of hierarchical structure of bone tissue composed of organic and inorganic materials [19], Copyright © 2018, The American Association for the Advancement of Science. The inorganics of the mineralized collagen fibrils themselves incorporate several nested structural motifs, listed as follows in decreasing order of complexity: mineral aggregates – stacks of platelets – platelets – acicular crystals. Collagen fibrils are composed of quasi-hexagonally packed microfibrils, each of which incorporates multiple staggered triple helices that in turn are formed from repetitive chains of amino acids. Ordered and disordered motifs of bone consist of mineralized collagen fibrils that are about 120 nm thick and build a continuous network

Among organic substances, non-collagenous proteins are produced by osteoblasts and constitute approximately 10% of bone organic matter [13, 14]. The organ matrix of bone tissue includes osteocalcin (OC), which accounts for 10% of the non-collagenous, osteopontin (OP), which inhibits mineralization, and bone sialoprotein (BSP), which is required for HAp nucleation. Although it accounts for a very small percentage, proteoglycans of the bone matrix affect the initial stages of bone growth. In particular, osteonectin (ON) and SPARC (secreted protein, acidic, and rich in cysteine), phosphorus proteins that interact with Col-I and HAp, are present in the matrix immediately adjacent to osteoblasts and osteocytes [15, 16]. They play an important role in the early stages of bone formation, in bone growth and regeneration, affecting osteoblast and osteoclast functions. In addition, the bone matrix is composed of various growth factors (bone morphogenetic proteins, fibroblast growth factors, platelet-derived growth factors, insulin-like growth factors, and transforming growth factor-β) that greatly influence the differentiation of preosteoblasts and stem cells into bone tissues [17, 18]. The bone tissue composed of the organic and inorganic materials mentioned so far is summarized in Fig. 2 from the atomic units to the macro-scale.

### Calcium-based biomaterials for bone regeneration

#### Calcium-based materials

Calcium (Ca) is an essential nutrient for living organisms. Most of the calcium ions are in existence by binding other ions in the form of biominerals [20]. Owing to their excellent biocompatibility, bioactivity, and biodegradability, there has been a continuous research on calcium-based materials in various biomedical applications leading to the development of synthetic methods. Among them, calcium phosphates (CaPs) are the main inorganic components of hard tissues [13]. The major CaPs (Table 2), nanostructured amorphous calcium phosphate (ACP), HAp, and calcium-deficient hydroxyapatite (CDHA), are synthesized for biological applications because they are stable under physiological conditions and are easily synthesized and prepared from aqueous solutions using relatively simple methods.

**Table 2:** Properties of the major CaPs obtained from aqueous solutions [21]

Name	Chemical formula	Ca/P ratio	Solubility at 25ºC, -log(*K*_*s*_)	Solubility at 25ºC (g L^−1^)	pH stability range at 25ºC
MCPM	Ca(H_2_PO_4_)_2_·H_2_O	0.5	1.14	~ 18	0.0–2.0
DCPD	CaHPO_4_·2H_2_O	1.0	6.59	~ 0.088	2.0–6.0
OCP	Ca_8_(HPO_4_)_2_(PO_4_)_4_·5H_2_O	1.33	96.6	~ 0.0081	5.5–7.0
ACP	Ca_*x*_H_*y*_(PO_4_)_*z*_·*n*H_2_O, *n* = 3–4.5; 15–20% H_2_O	1.2–2.2	^a^	^a^	~ 5–12
CDHA	Ca_10-*x*_(HPO_4_)_*x*_(PO_4_)_6-*x*_(OH)_2_ (0 < *x* < 1)	1.5–1.67	~ 85	~ 0.0094	6.5–9.5
HAp	Ca_10_(PO_4_)_6_(OH)_2_	1.67	116.8	~ 0.0003	9.5–12
FAp	Ca_10_(PO_4_)F_2_	1.67	120.0	~ 0.0002	7–12

*MCPM* monocalcium phosphate monohydrate, *DCPD (brushite)* dicalcium phosphate dehydrate, *OCP* octacalcium phosphate, *FAp* fluorapatite

^a^Metastable, no exact value due to various variables

Similar to CaPs, calcium carbonate (CaCO_3_) is an omnipresent and important biomaterial in biological systems and is widely used to study biomimetic processes. CaCO_3_ is spontaneously generated in the unstable amorphous calcium carbonate (ACC) phase, with two hydrated metastable forms and three anhydrous crystalline polymorphs in the order of their stability [22–24]. Calcium silicate (CaSi) is widely used as a bioceramic, bioglass, and bone cement, especially in orthopedic and oral-maxillofacial surgery. CaSi can completely transform into CaPs after soaking in a phosphate-concentrated solution because its *K*_sp_ is much higher than HAp [25].

To control the physicochemical properties of calcium-based materials, it is important to develop new processes that can precisely control the crystal structure and chemical nature of the materials [26]. The synthesis of nano sized inorganic compounds involves difficulties in controlling their structure, size and size distribution, crystallinity, and stoichiometry. Each preparation method significantly affects the formation of crystalline phases and structures in CaP biomaterials [26, 27]. Nanostructured CaPs with poor crystallinity are typically synthesized using a precipitation method at room temperature under mild conditions. Sonochemical, microwave-assisted synthesis, and hydrothermal methods have been continuously developed to increase crystallinity and control the morphology and structure of CaPs. A comparison of the major methods used to synthesis CaP is summarized in Table 3.

**Table 3:** A comparison of major methods for the synthesis of CaPs [20, 28]

Methods	Advantages	Disadvantages
Precipitation	- Simple setup - Low operating temperature - Convenient doping with other ions - Uniform structures - High production yield of pure product	- Poor crystallinity - Irregularly formed - Inhomogeneous in composition - Calcium deficient
Hydrothermal	- Good crystallinity - Convenient doping with other ions - Hierarchical structure	- Energy consumption - Low production yield of pure product
Sol–gel	- High homogeneity - High purity - Lower processing temperature	- Expensive raw materials - Difficulty of hydrolysis rate control - Long processing time - Cycles depending on sol viscosity

There are some other synthesis methods including high gravity, methods high-temperature pyrolysis, mechanochemical, microwave-assisted synthesis, precursor transformation, solution combustion, sonochemical synthesis, and spray pyrolysis

As mentioned earlier, CaPs are the main inorganic components that constitute hard tissues. Among these, HAp is the major mineral in vertebrate bones and teeth. Hydroxyapatite, the most thermodynamically stable crystalline phase of CaP in vertebrates, is the mineral that organizes bone [29]. For decades, HAp has become a subject of interest because of its excellent biocompatibility, an affinity for bio-organics, and high osteogenesis potential [30, 31]. It has been well established that HAp can promote bone regeneration through an osteogenic mechanism without causing local or systemic toxicity, and inflammation of foreign substances [32, 33].

### Synthesis methods of calcium phosphates

In the early days, studies focused on the chemical properties of synthesized HAp. In recent years, studies have been conducted on the actual HAp growth mechanism that constitutes the human body. As we shall see later, the inorganic CaPs in the actual bone tissue are indeed placed in association with organic matter in a unique crystalline state, not in a bulk state; therefore, the study of the size and shape of the CaP crystals during growth is essential. Over the past few decades, much effort has been made to control the size and shape of CaP crystals, including developing new strategies and modifying existing methods [26, 34].

**Fig. 3:**
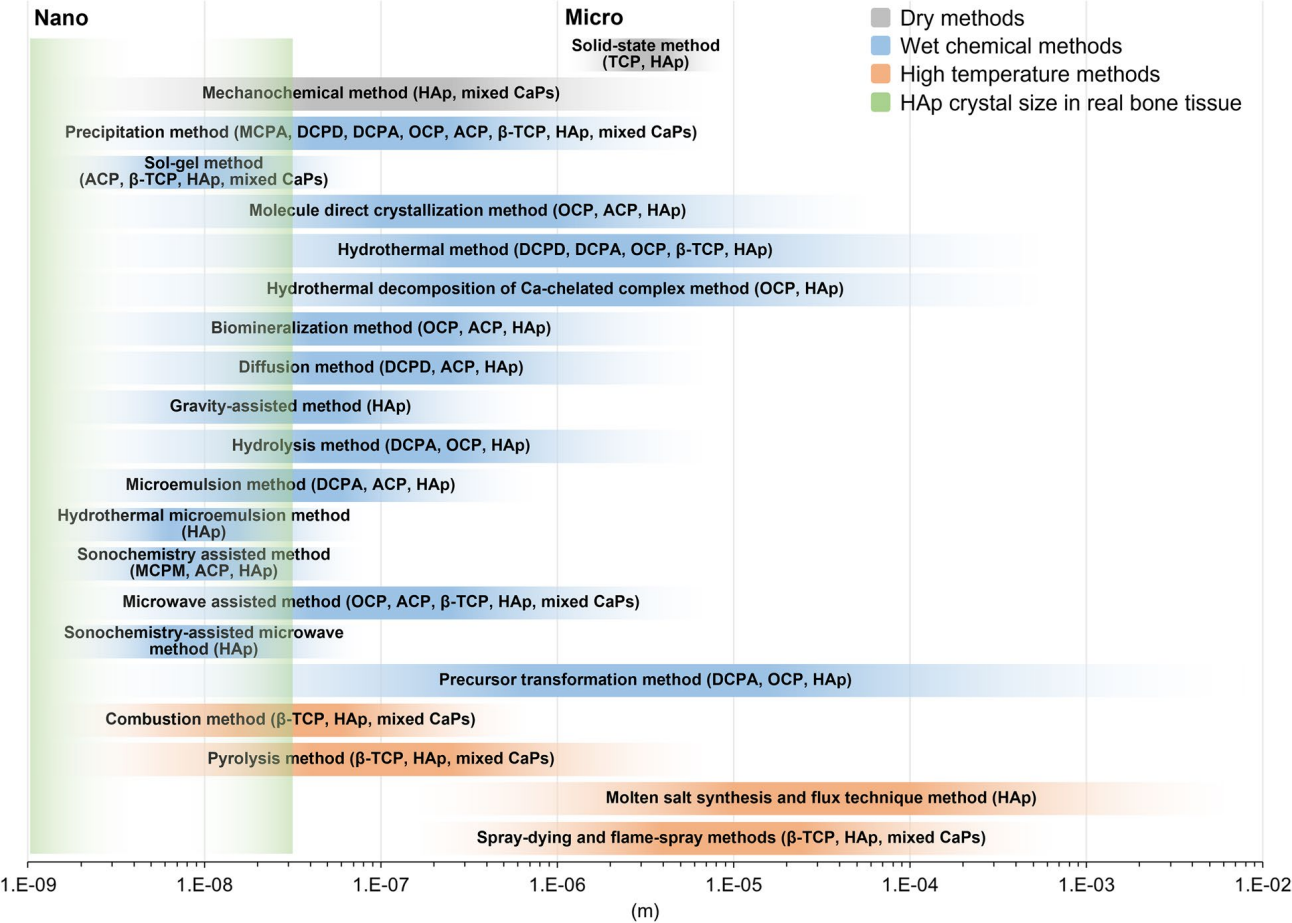
Summary and comparison of the size and size distribution of CaPs using various synthesis methods (solid-state method [27], mechanochemical method [38], precipitation method [39], sol–gel method [40], molecule direct crystallization method [41–43], hydrothermal method [44, 45], hydrothermal decomposition of Ca-chelated complex method [46, 47], biomineralization method [48–50], diffusion method [51, 52], gravity-assisted method [53], hydrolysis method [46], microemulsion method [54, 55], hydrothermal microemulsion method [55], sonochemistry assisted method [56–58], microwave assisted method [59, 60], sonochemistry-assisted microwave method [57], precursor transformation method [48–50, 61], combustion method [62, 63], pyrolysis method [64, 65], molten salt synthesis and flux technique method [66, 67], and spray-drying and flame-spray methods [64, 65].). TCP; tricalcium phosphate, DCPA; dicalcium phosphate anhydrous

Many synthetic methods mentioned above have been applied in many strategies to control the size of CaP crystals and agglomerates. The size of the CaPs is related to the choice of the synthesis method. The size and size distribution of CaP crystals, including the ion concentration of the precursors, raw materials for calcium and phosphorus, type and concentration of organic and inorganic additives [35], reaction temperature, and initial and final pH values, play an important role in regulation [36, 37]. The dry method, including solid-state synthesis and mechanochemical methods, is less expensive and capable of mass-producing highly crystalline CaP than compared to the wet chemical method. However, the crystal size in the dry method is relatively large, the purity of the phase is low, and it is difficult to control the crystal size, particle size, and shape. Wet methods include chemical precipitation, sol–gel, hydrothermal treatment, diffusion, hydrolysis, emulsion, biomimetic strategies, and microwave and ultrasonic chemistry methods, which depend on functional additives. Wet methods can produce CaP crystals with precise control of the size and shape of the raw materials containing various types of calcium and phosphate. In addition, it is said to be a fundamental technique for deeply understanding the mineralization method in the body and the nucleation, growth, phase change, and self-assembly pathways of CaP crystals in solution. However, it is difficult to synthesize pure CaP crystals in large quantities with narrow size distribution and agglomeration by the wet methods, and there is also the disadvantage that the process is complicated and time-consuming. High-temperature methods such as combustion, pyrolysis, molten salt synthesis, flux technology, spray-drying, and flame-spray methods are advantageous for synthesizing highly crystalline CaP crystals. However, these methods have the disadvantage that it is difficult to precisely control the morphologies of the CaP crystal because the secondary aggregates and chemical phases are generally mixed with the particles obtained at excessively high energy consumption and temperatures. Lin et al. summarized and compared various methods used to synthesize CaPs of different sizes, but the exact values were not directly addressed [27]. We refer to the table in reference and graphically show the relationship between the synthesis methods of CaPs and the size and size distribution of the synthesized powder (Fig. 3).

#### Conventional synthesis methods of calcium phosphates

##### Precipitation method

**Fig. 4:**
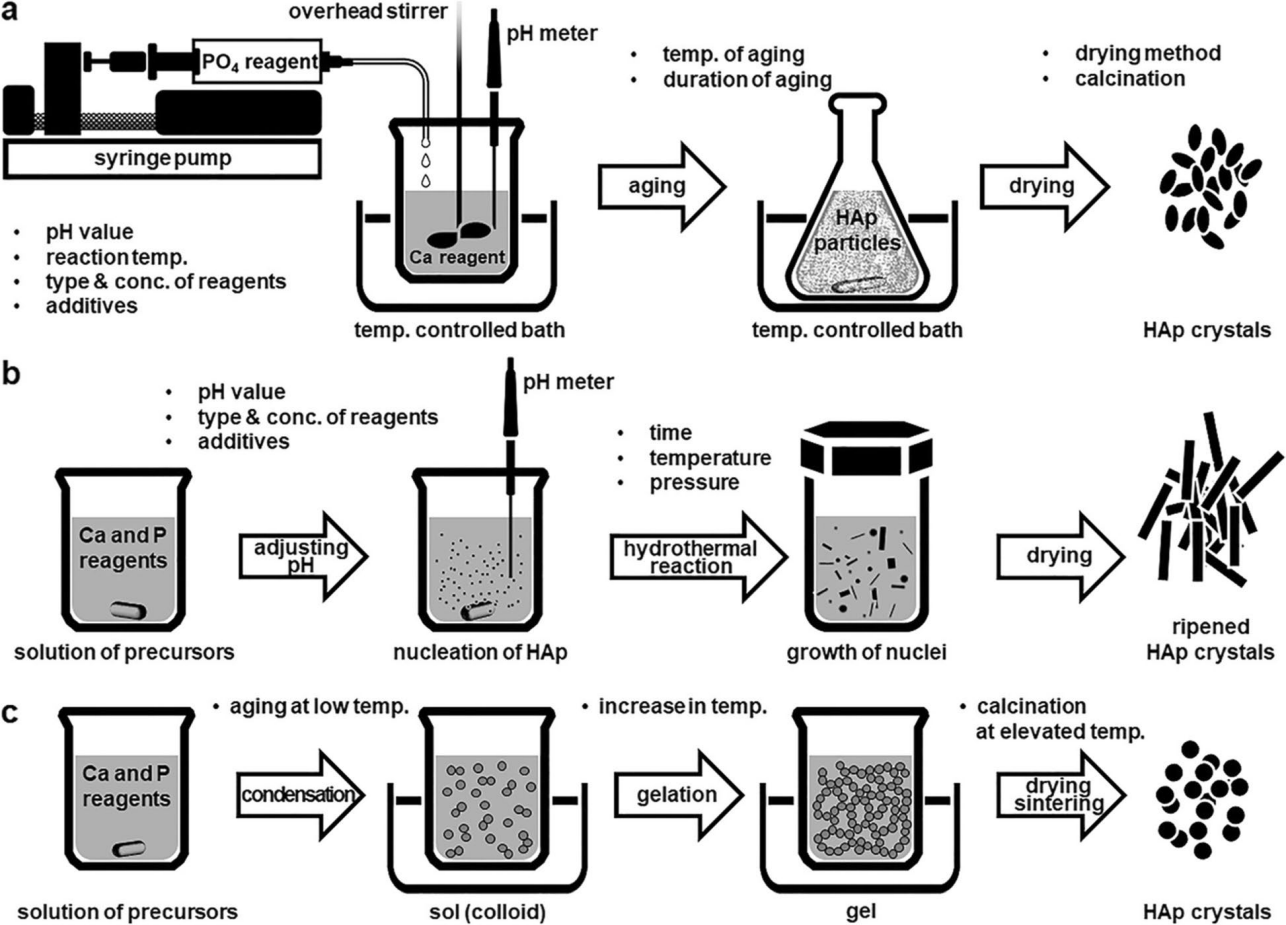
Preparation of HAp nanoparticles via (**a**) conventional chemical precipitation, (**b**) hydrothermal condition, and (**c**) sol–gel process

The precipitation technique is the simplest method for HAp synthesis. The resulting particle size, phase, and structure can be easily controlled by processing variables such as ion concentration, pH, solvent species, time, and additives [26]. The precipitation method is the main strategy for synthesizing CaP nanoparticles because of its convenience, flexibility, and simplicity. HAp is the least soluble in aqueous solution at room temperature and a pH of approximately 4.2 and is generally the most stable phase of CaP [68, 69]. Figure 4a shows a schematic process diagram of HAp precipitation with the suggested parameters that influence the properties of the synthesized powder. However, the prepared powders are generally nonstoichiometric and poorly crystallized without a regular shape [70].

##### Hydrothermal method

The hydrothermal method is generally one of the most common synthetic methods for HAp by reacting chemicals in an aqueous solution under high-pressure and high-temperature autoclaves or pressure vessels [26, 37]. Hydroxyapatite nanoparticles synthesized under hydrothermal conditions have proven to be relatively close to stoichiometry and highly crystalline [71, 72]. In addition, the Ca/P ratio and phase purity of HAp precipitates were notably improved as the hydrothermal temperature increased [73, 74].

HAp synthesized under hydrothermal conditions is typically characterized by forming rod-shaped crystals. Microcrystalline nuclei are formed in the supersaturated solution (reaction of ions) and grow continuously in their final shape and size (hydrothermal treatment) [75]. Figure 4b schematically illustrates these two steps [26]. The most prominent drawback of the hydrothermal method is the inability to control the shape and size distribution of particles. Temperature and pH are the most important factors influencing the structural and morphological properties of HAp nanoparticles. Mehdi et al*.* summarized recent results for synthesizing HAp nanoparticles under different pH conditions [76]. According to their results, a high pH value results in weak anisotropy (short nanorods) and, thus, in nearly isotropic growth (spherical nanoparticles). However, as the pH of the aqueous liquid decreases, anisotropic growth progresses; crystallites grow into a platelet morphology. In addition, more complex shapes, including 3D feathery structures, micro cubes, and microfibers, were obtained when the pH value dropped to 4, the pH at which other CaP phases dominate [76].

##### Sol–gel method

The sol–gel method was one of the first proposed methods for the wet synthesis of HAp. Sol–gel provides the advantage of molecular-level mixing of reactants, which improves the chemical homogeneity of the synthesized powder [77]. In the synthetic process, formation and fusion at low temperatures are other notable advantages of the sol–gel method over wet synthesis methods. According to in vitro studies, HAp synthesized by the sol–gel method can control the particle size at the nanometer-sized boundary, and the chemical structure during sintering. These features are better accepted for host bone tissue regeneration as they can also regulate the rate of biodegradation [78]. The generation of a secondary phase (generally calcium oxide, CaO) is the main shortcoming of the sol–gel method. The secondary CaO phase has proven to be cytotoxic; therefore, there have been attempts to wash the calcined powder using an acid solution or to modify the main procedure to remove the coexisting CaO phase [26, 79].

A typical sol–gel process involves mixing alkoxides in aqueous or organic solutions, followed by aging at room temperature, gelation, drying, and finally removing organic residues at high temperature from the dried gel, as shown in Fig. 4c. As with other wet methods, several precursors can be developed using a typical sol–gel process. In most cases, calcium diethoxide or calcium nitrate reacts with triethyl phosphite or triethyl phosphate in an aqueous or organic solution [80]. Hsieh et al*.* prepared nanocrystalline HAp using a sol–gel process and studied the effect of the gelation rate. They reported that a fast gelation rate for apatite formation leads to high CaO generation, whereas slow gelation produces less CaO that can be washed off with distilled water [79]. Recently, a non-alkoxide sol–gel synthesis process for HAp was developed without pH adjustment [78, 81]. The HAp nanoparticles produced in this process were sintered at 600 °C to obtain a nanoscale, low crystallinity, and carbonate apatitic structure similar to the minerals present in human bone tissue. These properties have shown the effects of increased surface area and bioresorbability in body fluids [78]. It has also been shown that HAp nano particles of different sizes can be obtained depending on the aging time [81]. It has been suggested that aging contributes to particle growth and aggregation.

### Biomineralization – mineralization in vertebrates

#### Mineralization mechanisms via classical and non-classical theories

According to conventional nucleation theory, crystal formation from the elements constituting a supersaturated solution requires a local change in the interacting ion concentration [82]. The favorable interaction of some ions at critical concentrations stabilizes the ion clusters such that the free energy obtained by ion dissociation is less than the free energy obtained by adding more ions to the clusters or nuclei for crystallization. This process can be thermodynamically induced by the free energy difference between the crystalline and liquid phases; however, it is dynamically controlled by the critical point of overcoming the energy barrier, including the surface energy.

**Fig. 5:**
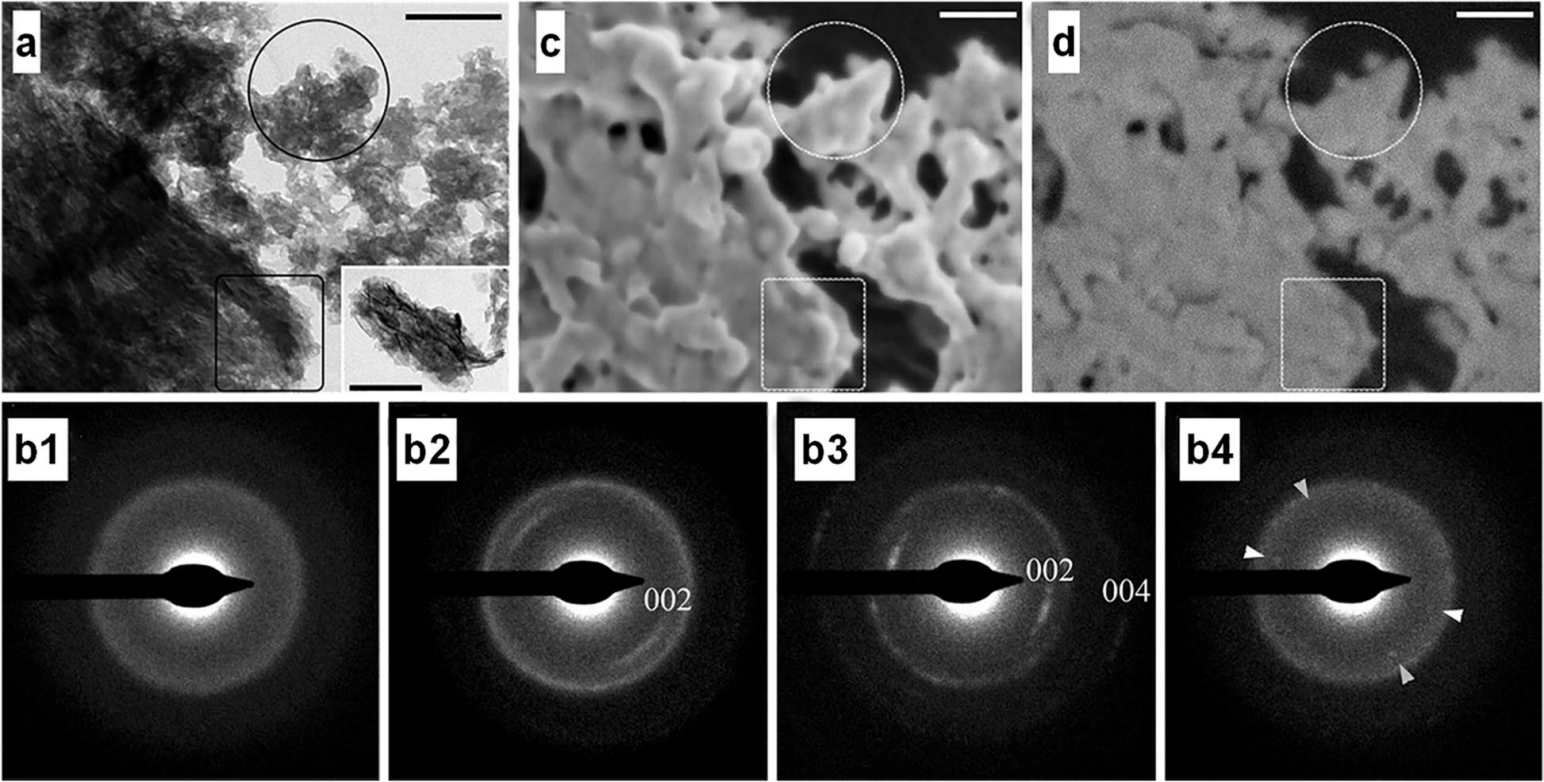
**a** Transmission electron microscope (TEM) image of mineral particle aggregates (scale bars 200 nm) and (**b**) the corresponding SAED patterns: (b1) particles enclosed in the circle indicate amorphous scattering of the diffusion ring. (b2) the marked rectangular area has poor crystalline diffraction, and (b3) the particles in the area shown in the interpolated image produced a clear crystalline diffraction pattern [(002) and second order (004) showing well defined reflections of apatite planes.] **(**b4**)** the encircled area taken after being stored at room temperature for a week. The appearance of diffraction spots with (002) plane (arrowheads) spacing suggests a transition to the crystalline HAp phase [85]. **c** Cryogenic-scanning electron microscope (Cryo-SEM) image of the same area (scale bar 100 nm). **d** The amorphous (encircled area) and crystalline (rectangular area) portions of the electron selective backscatter (ESB) image shows no significant difference in signal strength (scale bar 100 nm)

**Fig. 6:**
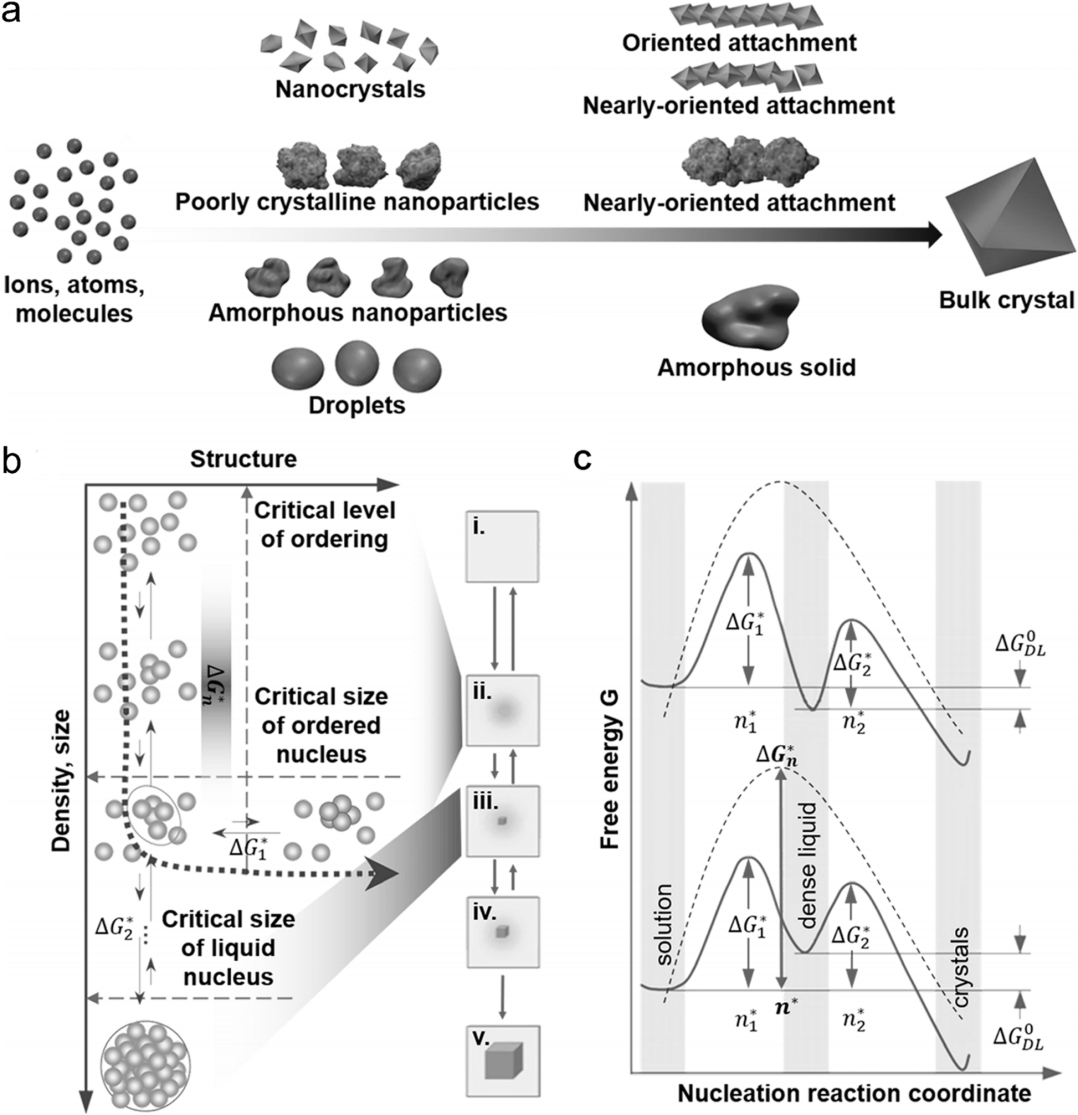
**a** Pathways to crystallization by particle attachment [83]. **b** The mechanism, consisting of two steps, shows the formation of crystal nuclei inside them [82]. (i) The supersaturated solution. (ii) The dense liquid. (iii) The critical cluster at *n*_2_*. (iv) The nucleation cluster and ensuing growth of the crystal. (v) the thoroughly formed crystal. **c** The path to the free energy change *ΔG* according to the two feasible models of the two-phase nucleation mechanism [82]

Crystallization in many systems can be induced by the broader attachment of more complex species than simple ions (Figs. 5 and 6) [83]. Many researchers have examined the current understanding of crystallization by particle attachment and have investigated the thermodynamics and kinetics that lead to crystallization. It is important to infer which of these pathways can occur in living organisms with bone tissue on the preferential basis. In particular, understanding the mechanism of biomineralization in vertebrates has long been a requirement. More than half of the biominerals from mineralization, such as CaP, are calcium-containing. Calcium phosphate-containing minerals make up the hard connective tissue of vertebrates, whereas CaCO_3_ is known to form the skeleton of invertebrates [84]. In practice, the theory of mineralization, as well as that in the normal vertebrate skeletal system (bone, cementum, dentin, and tendon tissues), simply mediating the pathological mineralization process, has not been fully understood. The pathway from amorphous to crystalline, when crystallized from ACP to HAp, and/or another crystal structure, occurs in the intermediate stage, which is theoretically the most reliable explanation.

Recently, as interest in collagen mineralization has increased, research findings in which crystal growth occurs in a specific orientation depending on the actual collagen fibril morphology have been proven through analytical techniques. For example, bone growth in zebrafish fins was characterized by determining the beginning and end stages of the formation and analyzing the orientation of the growing fins [85]. Selected area electron diffraction (SAED) pattern analysis of the beginning, middle, and end of the fin growth proved a crystalline phase with a specific growth direction through the amorphous phase (Fig. 5), as shown in Fig. 6a (bottom part), during the mineralization processes. The crystalline growth in a specific direction, parallel to the collagen fibril direction, was caused by the binding of the ‘nanocrystals’ and/or ‘poorly crystalline nanoparticles’ as shown in Fig. 6a (upper part). The results for the crystalline growth in the form of rods or needles can also be inferred. Organics induce crystallization, nucleation, and growth in an environment substantially similar to the human body. Indeed, for many organic [86] and inorganic [87, 88] crystals, nucleation models, such as pre-nucleated clusters, have been proposed. However, owing to the relatively small scale, it has not been possible to reveal the details of the cluster structure or the mechanism by which they are aggregated, and inherently hydrated. In addition, the effects of these clusters on the energy barriers that ultimately affect the pathway through the amorphous phase have not been studied [89].

Figures 6b and c schematically show the mechanism, consisting of two steps of crystal formation and a path to the free energy change *ΔG* according to two feasible models of the two-phase nucleation mechanism [82]. Inorganics in the human body are composed of specific ions; therefore, it is very useful to deduce the mechanism based on existing theory. Vekilov *et al*. explained nuclei formation in a dense liquid phase, as shown in Fig. 6b [82, 90, 91]. This mechanism involves the formation of dense liquid clusters, and crystal nuclei can form inside these clusters. Figure 6c shows the top curve when the dense liquid was unstable and *ΔG*_DL_º > *G*_SS_ (*ΔG*_DL_º: standard free energy of the formation of the dense liquid. *G*ss: free energy of the supersaturated solution). If the dense liquid is stabilized by introducing an external interface* ΔG*_DL_º < 0, a lower curve can be applied. *ΔG*_1_* is a barrier to forming dense liquid clusters and *ΔG*_2_* is a barrier to crystalline nucleation inside the dense liquid. Wolf *et al*. demonstrated the mechanism of crystal nucleation from a dense liquid precursor in a CaCO_3_ system [92]. They showed floating CaCO_3_ solution droplets between a piezoelectric vibrator that produced an acoustic wave and a concentrically adjusted sonic reflector. Calcium carbonate is homogeneously formed in an amorphous liquid-like state at neutral pH without any stabilizing polymer or additive. This stability in an amorphous liquid-like state at neutral pH can be closely related to the various carbonate groups present in the process, such as carbonates, bicarbonates, and non-dissociated carbonates. The formation of an amorphous liquid-phase mineral precursor was confirmed to be a hallmark of the true homogeneous formation of CaCO_3_ itself, and the resulting primary particles also confirmed that it acts in the second stage as a template for the crystallization of calcite. Ultrasonic trapping is an excellent method for the real-time analysis of nucleation, crystal growth, and phase separation processes with minimal disruption and artifacts caused by solid-phase boundaries. These results concluded that acoustic levitation provides a reliable condition for studying uniform precipitation reactions.

**Fig. 7:**
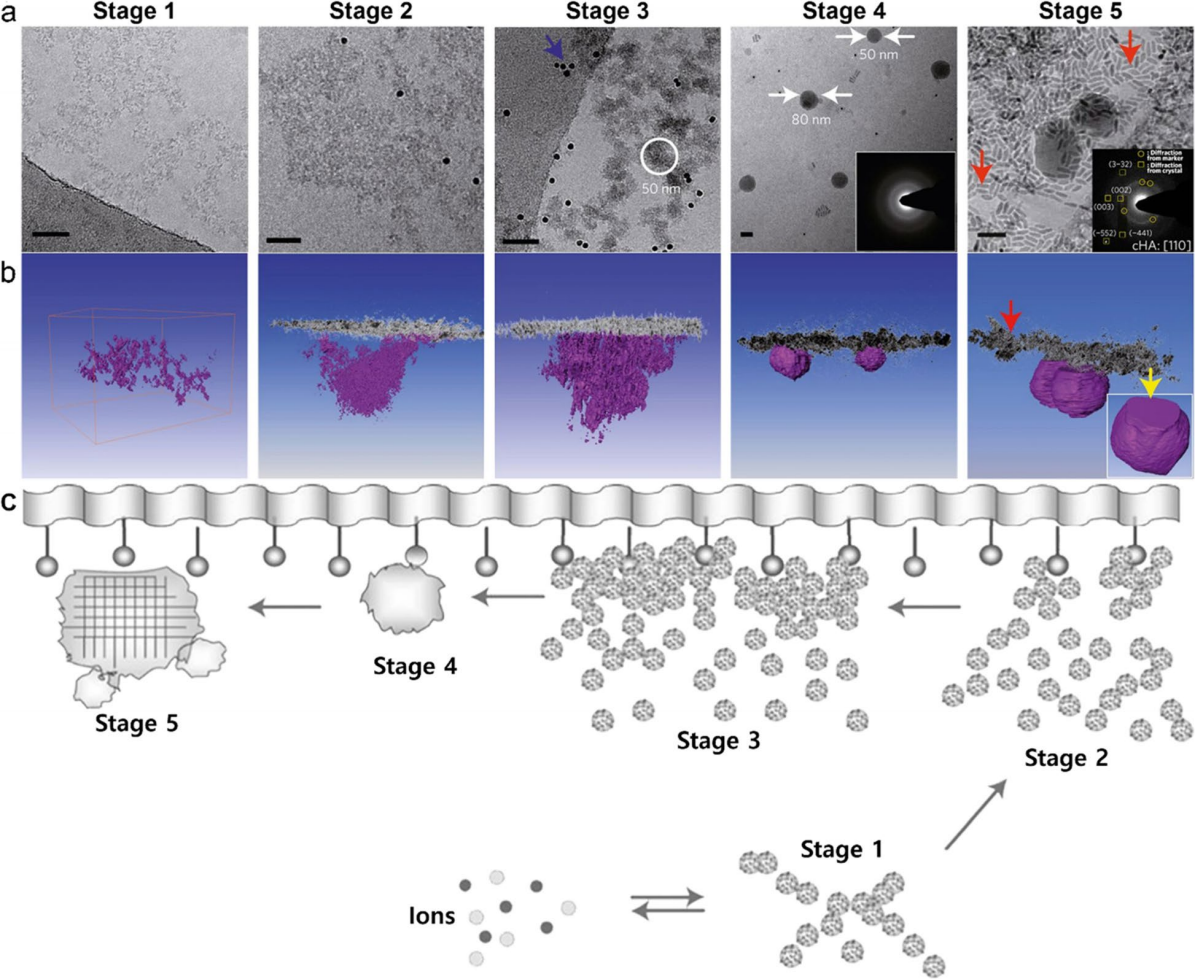
Mineralization stages. **a** Two-dimensional projection images and (**b**) three-dimensional visualizations of tomograms. Stage 1 is in the absence of a monolayer, and stages 2–4 are that the inset SAED pattern in (**a**) shows that the spherical particles on the monolayer are amorphous phases. Finally, stage 5 is that the inset SAED pattern in (**a**) can be indexed as cHAp with a [110] zone axis. The yellow arrow in (**b**) indicates the preferred nucleation plane (110). The red arrows indicate the markers, and the blue shows the Au beads. Scale bars, 50 nm. **c** Surface-directed mineralization stages of CaP from SBF at 37 °C. Stage 1 represents the loose aggregation of the prenucleation cluster in equilibrium with the ions of the solution, and stage 2 shows that the nucleated cluster aggregates in the presence of a monolayer so that the loose aggregate still exists in the solution. Stages 3 and 4 show cohesion leading to densification near the monolayer, indicating the nucleation of amorphous spherical particles only on the monolayer surface, and the last 5 stage shows the development of crystallinity due to oriented nucleation directed by the monolayer [95], Copyright © 2010, Nature Publishing Group

Posner *et al*. suggested that biological apatite with amorphous constituents, Ca_9_(PO_4_)_6_ and ion clusters of Ca and PO_4_ with 0.9−1.0 nm diameter can be stable as prenucleation clusters [93, 94]. Recent studies have shown that stable nuclei of CaP, as predicted, can be observed in simulated body fluid (SBF) at physiological temperature [95, 96]. It is suggested that the presence of nuclei surfaces resulted in composition and structural changes that allowed the dense packing of clusters and ensuing fusions to form ACP and eventually HAp crystals (Fig. 7). It was also confirmed that mineralization from SBF transpires exclusively via heterogeneous nucleation on the monolayer surface. This observation also distinctly demonstrates that HAp crystallization from SBF at body temperature in the presence of arachidic acid monolayer proceeds through a multi-step process involving the nucleation and aggregation of pre-nucleated clusters, as shown in Fig. 7a and schematically in Fig. 6b. This study makes it possible to discriminate between the different stages before amorphous nucleation (before stage 4). In the first stage, pre-nucleated clusters in the SBF aggregate and form a stable, loosely connected free-floating network in the solution. In the second stage, the part of the cluster aggregate that comes into contact with the organic film begins to be densified by adjusting the adjacent packing of the cluster. In the third stage, this densification process continues, leading to defined domains of intimately related communities up to 50 nm in diameter attached to the monolayer and further fusion to form monolayer-suspended ACP particles. These nanoparticles grow further and generate crystallinity, producing type B apatite spherical crystals (Type B apatite will be described in Section Convergence Theory from Biology and Materials Science.). Crystallization is induced by monolayers and alignment of the crystallographic *c*-axis along the nucleation surfaces after specific nucleation (110). Higher concentrations of carbonate affect spheroidal morphology during the growth of carbonated hydroxyapatite (cHAp) [97]. The typical plate-like morphology of the final HAp crystals can be explained by the gradual reduction of carbonate ions in SBF by ion consumption and CO_2_ gas release [95].

Studies on nucleation and crystal growth under biological conditions, such as in the human body, are under way. Many studies have been conducted to incorporate specific proteins and/or biological factors into nucleation and crystal growth, considering various body environmental variables. Biomineralized crystals are archetypally formed into organic matrices, with precise control of protein synthesis mechanisms. The primary amino acid sequences of the proteins (BSP, ON, OP, and OC) sometimes contain dense aspartic (Asp) and glutamic acid (Glu) residues, which are known to have a high affinity for Ca ions [98–101]. Yu et al*.* focused on amelotin, which has been previously described as an enamel matrix protein that plays an essential role in the biomineralization of dental enamel [102, 103], characterized by in situ analysis [104]. They demonstrated a phase transfer process using amelotin and the potential serine phosphorylation motif Ser-Ser-Glu-Glu-Leu (SSEEL), a common component of several proteins in the phosphoprotein family that binds to secreted calcium [105]. They also proposed a mechanism for rapid dissolution and recrystallization/reorganization during the active SSEEL motif-Ca^2+^ complex phase transformation after the secondary structural change of amelotin. Different steps affect the HAp growth mechanism, depending on whether the proteins are hydrophilic or hydrophobic. In hard tissue with extracellular mineralization, hydrophobic molecules generally produce a space-filling system, whereas hydrophilic molecules are known as sites for nucleation and thus mineralization [106, 107]. However, little is known about the interactions between proteins in the final HAp crystals.

**Fig. 8:**
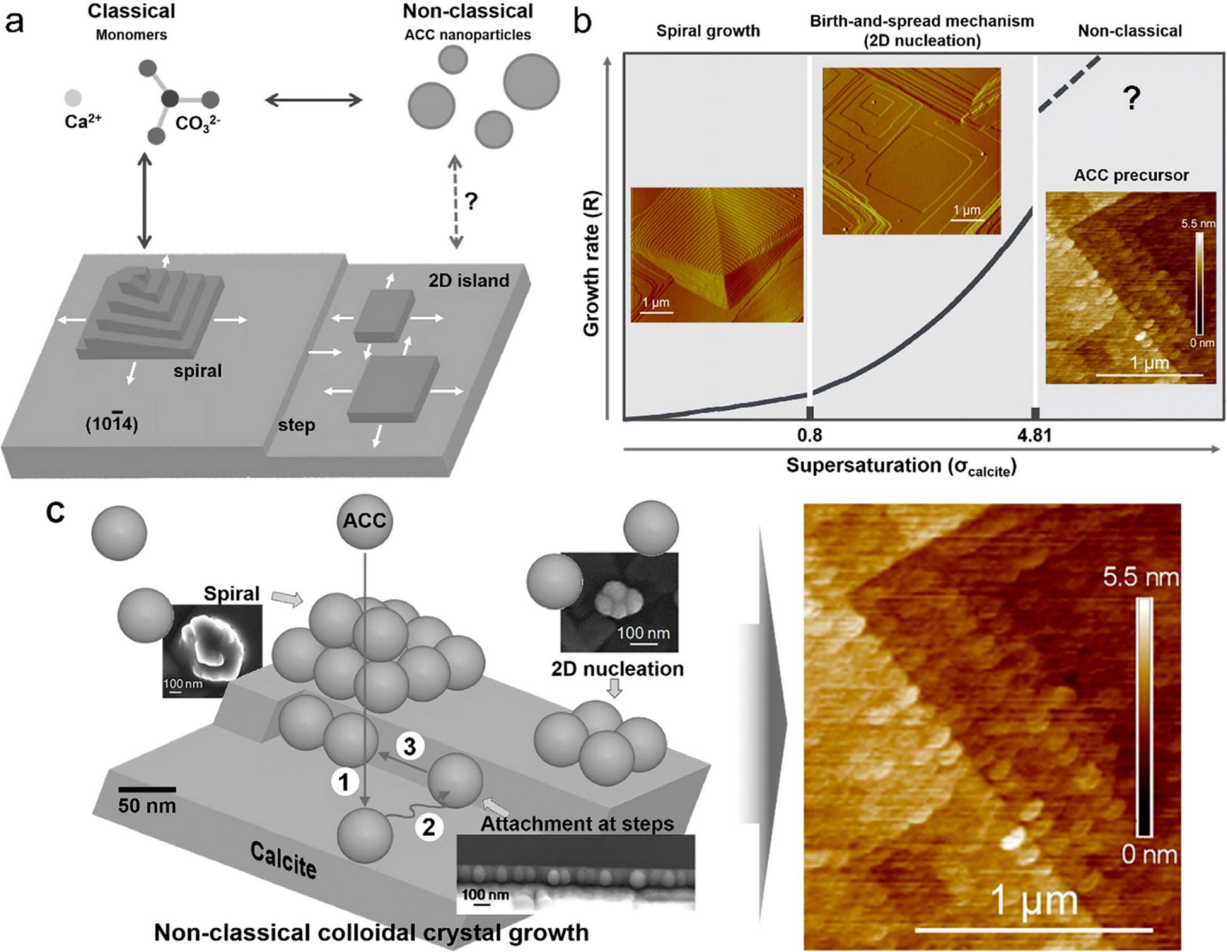
ACC nanoparticle precursors and calcite crystal growth. **a** Calcite growth via classical and non-classical pathways. **b** Growth rates, *R* vs supersaturation, *σ*_calcite_. Inset show in situ AFM deflection images of spiral growth and birth-spread model, and an image of calcite growth through ACC attachment along stages according to the non-classical mechanism of particle-mediated growth from a colloidal dispersion at the interface between calcite and solution. **c** Nanoparticles on (10.4)_calcite_. ACC nanoparticle bulk diffusion (1), surface diffusion (2), and final diffusion/attachment to a corner (3) [108], Copyright © 2016, American Chemical Society

Although recent works emphasize the importance of non-classical crystallization pathways involving amorphous precursors, explicit proof of the actual mechanism of CaP crystal growth through amorphous phases is lacking, and research is steadily progressing in this area [108]. Rodriguez-Navarro et al. reported crystal growth in solutions at room temperature using in situ atomic force microscopy (AFM) of calcite [108, 109]. The results showed direct nanoscale proof that amorphous calcite crystals can grow non-classically by a layer-by-layer process involving the attachment of ACC nanoparticles (Fig. 8) [108]. The formation and attachment of ACC nanoparticles at high supersaturation is a realistically feasible classical crystal-growth mechanism for faceted calcite crystals. This mechanism can challenge the current understanding of crystal growth in solution and the reflection of classical ionic-mediated crystal growth on the nanometer scale [110, 111]. Previously, ACC nanoparticle attachment that can form 2D islands and/or macro spirals on the calcite surface by surface diffusion and final incorporation with dehydration/restructuring has already been observed. Calcite crystal growth can proceed through classical pathways, such as ion integration, and non-classical pathways, such as nanoparticle adhesion, depending on supersaturation. In terms of classical (ion incorporation) versus non-classical (nanoparticle attachment) mechanisms, both growth routes follow the same helical and 2D nucleation processes, despite the size difference between the building units. The molecular forms of CaCO_3_ biominerals and their commonly observed biomimetic counterparts can arise from colloidal crystal growth through the attachment of ACC nanoparticles to crystalline CaCO_3_ substrates. However, these nanometer-scale properties are preserved only when they are converted to calcite in the presence of organic molecules, such as polyacrylic acid (pAA). ACC conversion to calcite can occur through an interface-coupled dissolution–precipitation mechanism. This mechanism typically results in a pseudomorphic feature. If precipitation occurs after complete dissolution, retaining the nano-granular properties of the ACC growth layer, no morphological substitution of ACC by calcite would have occurred. This pseudomorphism, which leads to the nano-granule characteristics shown in their study, is preserved only in the presence of pAA. In the absence of pAA, calcite continued to grow even after conversion from ACC to calcite and lost ACC nanogranular properties (Fig. 8b). These results can be interpreted in various ways based on existing mechanisms; however, further research is required.

**Fig. 9:**
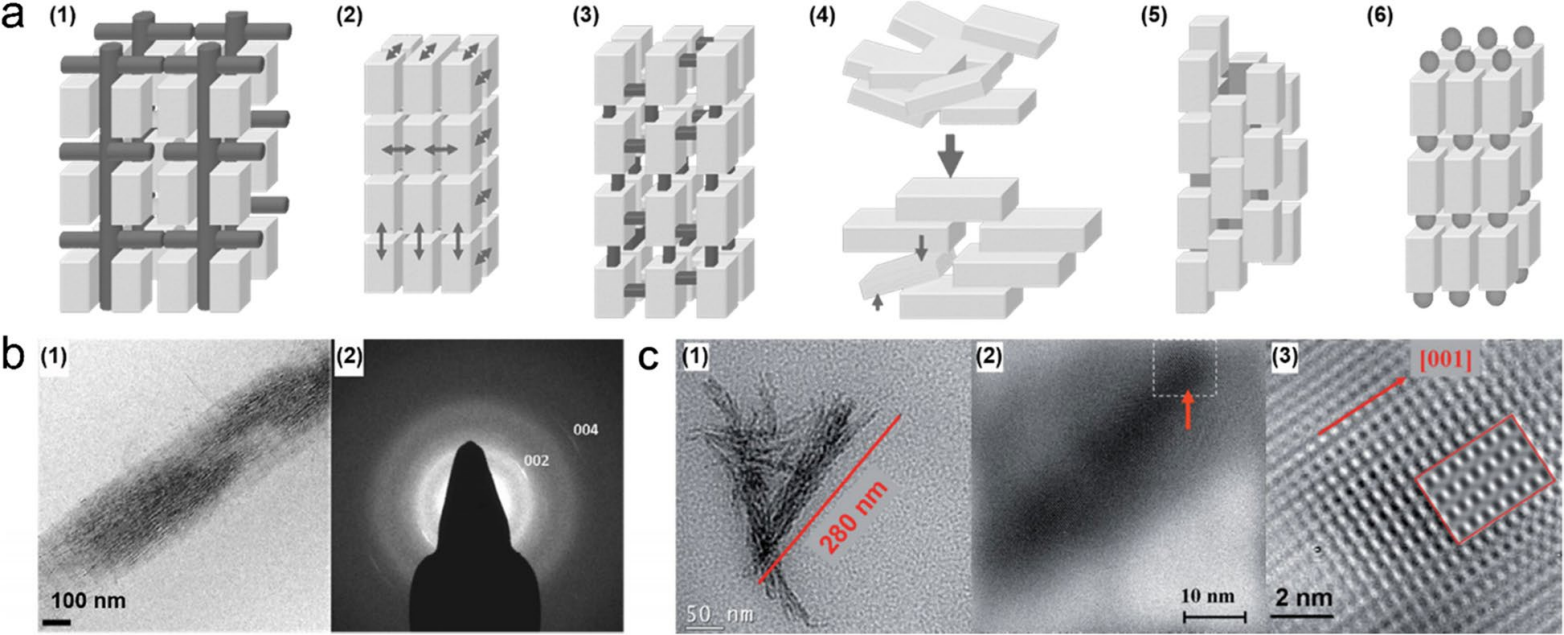
**a** Mechanism of mesocrystals; (1) alignments by the organic matrix and (2) physical forces, (3) crystalline bridges by epitaxial growth and secondary nucleation, (4) alignments by spatial constraints, (5) oriented attachment, and (6) face selective molecules [113]. **b** Cryo-TEM (1) image and (2) SAED pattern of the mineralized collagen fibril in the presence of pAsp [117]. **c** (1) TEM image of mineralized triple-helix protein molecule, (2) and (3) filtered and zoomed images of mineralized triple-helix protein molecules [123], Copyright © 2009 WILEY‐VCH Verlag GmbH & Co. KGaA, Weinheim

Several studies have been performed to understand the mechanism by taking advantage of advanced nanometer-analysis methods [88, 112]. As previously mentioned, vertebrate hard tissue is a hybrid nanocomposite composed of collagen and HAp. Collagen macromolecules strongly control the nucleation and growth of HAp, so there is a close orientational relationship between them. Hard tissues in the human body are mesocrystalline materials, kinetically stabilized nano-structured crystals that integrate crystallographically aligned nanocrystals’ properties. Mesocrystals are a fascinating class of nano-structured crystals [83, 113, 114]. Strum et al. identified six growth pathways for mesocrystals, schematically shown in Fig. 9a [113]. Among them, nanoparticle alignment is facilitated by an organic macromolecular matrix in the human body’s hard tissue. Many unknown biological factors in the human body successfully generate organic–inorganic nanocomposites in hard tissue, acting as functional factors and exhibiting hierarchical structures [107]. As commonly known, the bone and dentin hard tissues are composed of collagen and cHAp. The *c*-axis in the HAp crystal structure is nearly parallel to the long axis of the macromolecules that comprise collagen and is known to be similar to the mesocrystalline structure of biominerals at a hierarchical level [115, 116]. Biomineralization of collagen fibrils was accomplished by the nucleation and crystallization of HAp nanoparticles from modified SBF (m-SBF) containing polyaspartic acid (pAsp) [117–119]. These studies showed that the nanocomposite structure has a strong relationship with the structure of the bone tissue. Plate-like apatite nanocrystals (2–5 nm in thickness, 15–55 nm in length, and 5–25 nm in width) represent a desirable crystallographic orientation reflecting (*c*-axis of HAp is parallel to fibril elongation) the mesocrystalline structure (Fig. 9b).

Another example of successful biomimetic mineralization of analogs of materials is the FAp-gelatin nanocomposite system obtained by double diffusion [120]. This study was performed based on atomistic simulations of the design of crystallized FAp structures by binding the corresponding ions to the triple helix of collagen molecules [121, 122]. Ca_3_F-triangles are preferably oriented in a plane perpendicular to the long axis of the triple-helical protein [121]. Fluorapatite nanoplatelets covered collagen fibrils in a mosaic arrangement, where the crystallographic *c*-axis of FAp was parallel to the long axis of the fibrils (Fig. 9c), similar to the bone structure.

#### Collagen and non-collagenous proteins for mineralization

Fibrous proteins are abundant outside cells, forming an extracellular matrix that helps cells bind and form tissues. These proteins are secreted into the periphery by cells and assembled into sheets or long fibrils. Collagen is the richest fibrous extracellular protein in animal tissue. The collagen molecule comprises three long polypeptide chains containing the non-polar amino acid glycine at its third position. This regular structure allows the chains to wrap around each other, causing glycine to form a long, regular triple helix in the core. Many of these collagen molecules combine side by side and end-to-end to create an overlapping arrangement called collagen fibrils, which is very strong and helps to keep tissues together. Collagen belongs to a diverse family of proteins. More than 40 different collagen genes in mammals encode a variety of collagens that support the structure and function of various tissues. Collagen is the main protein in bones, tendons, and skin, making up 25% of the total protein mass in mammals, and more than any other type of protein. Col-I makes up 90% of the collagen in the body [124]. In bone tissue, Col-I accounts for approximately 95% of the total collagen content in bone and 80% of the total protein present in bone [125]. It can be said that Col-I constitutes the largest amount of bone tissue.

Among the cells in particular, differentiated osteoblasts produce and secrete proteins that constitute the bone substrate [126]. The main protein secreted by differentiated osteoblasts was Col-I. Initially, the protein is secreted at the amino-terminal and carboxyl ends of the molecule in the form of precursors containing peptide extensions, and propeptides are removed by proteolysis. After this process, further extracellular treatment results in the production of mature three-chained Col-I molecules, individually assembled into collagen fibrils and interconnected by forming pyridinoline crosslinks unique to the bone [127].

The collagen fibers formed in this manner provide a framework known as the extracellular matrix (ECM), in which inorganic crystals nucleate and grow in bone tissue. Although ECM influences the ultimate structure and orientation of HAp crystals in bone tissue, it cannot initiate HAp mineralization, even if the body fluid is supersaturated for HAp. HAp nucleation is initiated primarily by negatively charged phosphorylated non-collagenous proteins (NCPs; OP, BSP, dentin matrix protein 1 (DMP1) and dentin phosphophoryn (DPP)) associated with the ECM consisting of Col-I. These proteins enhance local supersaturation to a level sufficient to form critical-sized nuclei that can grow into HAp crystals by attracting the major constituent ions of HAp, Ca^2+^ and PO_4_^3−^, through the charged amino acid domain [128]. Among the amino acids, negatively charged amino acids such as Asp, Glu, and phosphoserine (PSer) are abundant in the acidic domain of NCPs, which are involved in the mineralization of HAp in hard tissue. In addition, Landis et al*.* showed that charged amino acids are in the hole zone of collagen, where HAp forms nuclei in fibrils [129]. These amino acids appear important for interaction with Ca^2+^ and PO_4_^3−^ ions, which are required for HAp precipitation.

**Fig. 10:**
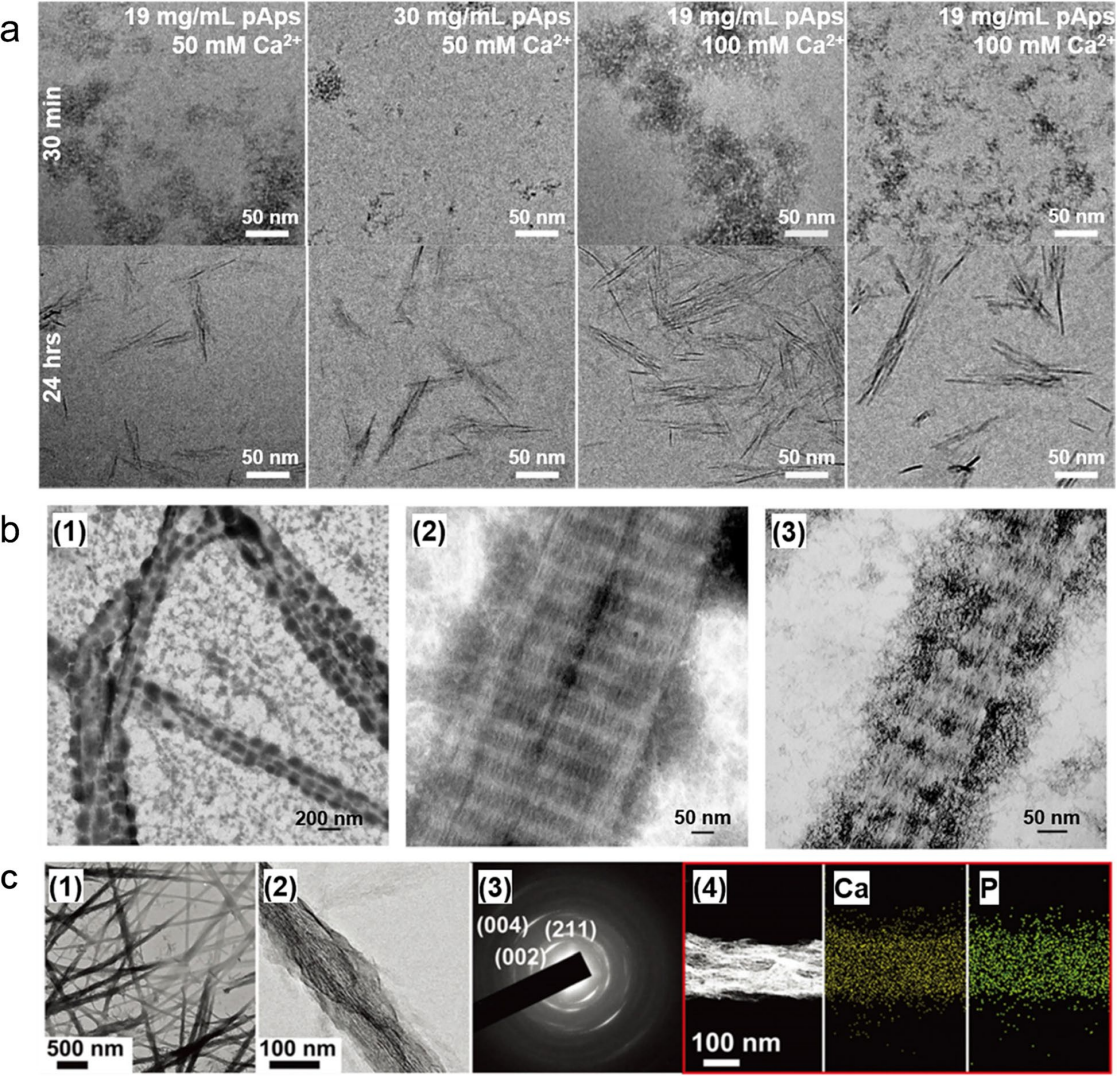
TEM images based on pAsp, is the most abundant amino acid group in NCPs and is used as a basic element in mineralization studies. **a** Cryo-TEM images according to pAsp for ACP precursor and Ca^2+^ concentration and time for crystal growth [130], Copyright © 2017, American Chemical Society. b Uranyl acetate staining TEM image of ACP mineralized reconstituted Col-I fibrils made anionic by binding with pAsp [132]. (1) The large and electrodense particles condensed on the fibril surface are ACP coacervates due to the electrostatic interaction between the polyvalent cation and the polyanionic electrolyte. (2) Adsorption of these electrodense particles slows the penetration of CaP. (3) In the maturation stage, the surface coacervate transforms into extrafibrillar CaP crystals, while mineralization intrafibrillar results in the formation of less heavy mineralized fibrils. **c** Mineralization of collagen fibrils in the presence of pAsp [134], © 2018 WILEY‐VCH Verlag GmbH & Co. KGaA, Weinheim. (1) Partially mineralized collagen fibers. (2) Enlarged image of mineralized collagen fibrils. (3) The SAED pattern in panel (2) is consistent with HAp and indicates oriented crystallization. (4) Elemental mapping of mineralized collagen fibrils

Among the factors that have amino acids, studies on negatively charged Asp, particularly and based on this effect, are being conducted steadily. Krogstad et al*.* observed the structural evolution and kinetic mechanisms of pAsp-induced CaP mineralization using pAsp, calcium, and phosphate concentrations as variables (Fig. 10a) [130]. Cryo-TEM demonstrated the following steps for CaP mineralization: (1) the formation of aggregates of pAsp-stabilized CaP spherical nanoparticles, (2) crystallization of nanoparticles, (3) orientation of nanoparticles to nanorods, and (4) crystallization of nanorods. The intermediate aggregate size and reaction kinetics were highly dependent on the pAsp concentration, but the particle size was not dependent on the pAsp concentration. This study showed which stages of pAsp affect the mechanism of CaP mineralization. Quan and Sone studied the effect of pAsp chain length on HAp deposition in demineralized periodontal tissues to confirm the role of pAsp in collagen mineralization [131]. The results confirmed that pAsp chain length exerted the effect of pAsp in mediating intrafibrillar mineralization.

Polyaspartic acid, which has amino acid domains, and is particularly negatively charged, has been applied in many other studies with objective confidence in the generation of precursors, which is the first step in the stable growth of inorganic crystals on the collagen matrix. Niu et al*.* established a new model for mineralizing collagen fibers in the presence of pAsp and other polyelectrolytes. Electrostatic attraction influences polyelectrolyte-directed intrafibrillar mineralization using polycation- and polyanion-directed intrafibrillar mineralization (Fig. 10b) [132]. Xu et al*.* conducted a theoretical calculation and simulation to improve the clarity reality of the sub-nanoscale nucleation mechanism of CaP in the collagen matrix in the mineralization of skeletal tissue [133]. These studies also analyzed mineral deposition and mineralization using pAsp and Glu in the computational process, providing atomic-level insight into the nucleation mechanism of inorganic crystals in the collagen matrix. Shao et al*.* experimentally demonstrated that citric acid molecules significantly reduced the interfacial energy between the collagen matrix and the CaP precursor and enhanced the wetting effect in the initial mineralization step, sequentially promoting the formation of CaP in Col-I fibrils (Fig. 10c) [134]. This study also provided results using pAsp as a basic additive in the CaP precursor formation process, which is the initial stage of mineralization after Col-I self-assembly, and then using citric acid as a variable.

#### Convergence theory from biology and materials science

In living organisms, many ions exist at different concentrations depending on the specific temperature and pH range. Based on these findings, many different solutions with different blood plasma concentrations have also been prepared and used in research. As shown in Table 4, ongoing studies control the specific ion concentrations in the known blood plasma. Contrary to the reasons mentioned earlier for SBF (related to bioactivity) [135], recent studies have been conducted to vary the concentration of ions in these solutions to obtain the desired crystalline form and size for mineralization. There is also a great deal of research using supersaturated SBF for the surface modification of medical devices and other biomaterials, such as dental implants, or for powder synthesis by precipitation [136, 137]. However, these studies may not be adequate to determine the mechanism because there may be sufficient interpretation differences to identify new bone growth and regeneration mechanisms.

**Table 4:** Ionic concentration (mM) of blood plasma and SBF studied to date

Formulation	Na^+^	K^+^	Mg^2+^	Ca^2+^	Cl^−^	HCO_3_^−^	HPO_4_^2−^	SO_4_^2−^	Buffer
Blood plasma [138]	142.0	5.0	1.5	2.5	103.0	27.0	1.0	0.5	-
Original SBF [33]	142.0	5.0	1.5	2.5	148.8	4.2	1.0	0	Tris
Corrected (c-SBF) [139]	142.0	5.0	1.5	2.5	147.8	4.2	1.0	0.5	Tris
Tas-SBF [140]	142.0	5.0	1.5	2.5	125.0	27.0	1.0	0.5	Tris
Bigi-SBF [138]	141.5	5.0	1.5	2.5	124.5	27.0	1.0	0.5	HEPES
Revised (r-SBF) [141]	142.0	5.0	1.5	2.5	103.0	27.0	1.0	0.5	HEPES
Modified (m-SBF) [141]	142.0	5.0	1.5	2.5	103.0	10.0	1.0	0.5	HEPES
Ionized (i-SBF) [141]	142.0	5.0	1.0	1.6	103.0	27.0	1.0	0.5	HEPES

*Tris* tris(hydroxymethyl)aminomethane, *HEPES* 4-(2-hydroxyethyl)-1-piperazineethanesulfonic acid

In an environment where various ions are present, most of the body is composed of carbon-based organic bonds. However, bone tissue is characterized by the growth of inorganic substances that bind to specific ions and structures. The ions present in our bodies continuously react chemically with each other. In addition, ions enter the mineral synthesis stage at any moment. For this process, we also need to know the binding force between the ions and how to maintain the concentration of the ions that cause the reaction. As shown in Tables 5 and 6, hard tissues in the human body, namely enamel, dentin, and bone apatite, are known to have different crystallinities, mainly owing to the concentration of trace ions (e.g., Mg, N, CO_3_, and HPO_4_) [42]. Since crystallinity also affects the solubility of apatite, the synthesized CaP powder used in the clinical field also emphasizes crystallinity. There is a significant effect of incorporating CO_3_ or Mg ions on the size and morphology of apatite crystals, and the degree of crystallinity [42]. Alternatively, proteins or other elements (e.g., pyrophosphate and citrate) may also inhibit the growth of biological apatite crystals [42, 97].

**Table 5:** Physical properties of representative hard tissues [142, 143]

	**Crystallographic**				
	Lattice parameters (Å)		Crystallite size (nm, avg.)	Crystallinity index, *b*	Ignition products (800 ºC)
	*a*-axis	*c*-axis			
Bone	9.418	6.884	2.5 × 0.3	33 − 37	HAp
Dentin	9.419	6.880	2 × 0.4	33 − 37	HAp + β-TCMP
Enamel	9.441	6.880	33 × 3	33 − 37	HAp + β-TCMP

Lattice parameters (± 0.003 Å); in substance, the values vary with age

HAp: *a*-axis = 9.422 Å, *c*-axis = 6.882 Å, crystallinity index = 100

*TCMP* magnesium substituted β-TCP

**Table 6:** Composition (wt%) of representative hard tissues [142, 159]

	**Composition** (trace elements: Zn^2+^, Cu^2+^, Fe^3+^, Sr^2+^, etc.)								
	Ca^2+^	PO_4_^3−^	Na^+^	Mg^2+^	K^+^	CO_3_^2−^	F^−^	Cl^−^	P_2_O_7_^4−^
Bone	34.8	15.6	0.9	0.72	0.03	7.4	0.03	0.13	0.07
Dentin	35.1	16.2	0.6	1.23	0.05	5.6	0.06	0.01	0.10
Enamel	36.5	17.7	0.5	0.34	0.06	3.5	0.01	0.30	0.02
	Total inorganic (mineral)			Total organic			Absorbed H_2_O%		
Bone	65.0			25.5			10.0		
Dentin	70.0			20.0			10.0		
Enamel	97.0			1.5			1.5		

Several studies have reported the nature of CO_3_ incorporation into biological apatites. The larger *a*-axis dimension of the phosphorus in the body compared to pure HAp is due to the CO_3_-for-OH substitution (type A) in these apatite [144]. Usually, this type of substitution is the result of apatite synthesized at high temperatures, showing extended *a*-axis and contracted *c*-axis dimensions compared with pure HAp [145]. In contrast, studies on apatite synthesized at low temperatures (RT − 100 °C), such as precipitation or hydrolysis methods, show partial CO_3_-for-PO_4_ substitutions (type B) coupled with partial Na-for-Ca substitutions [97, 146]. The results showed a contracted *a*-axis and an expanded *c*-axis compared with apatite and without CO_3_. Therefore, the study of the effect on the crystallinity of HAp under actual body temperature and other conditions, should be continually studied, before the bio-mechanism can be identified.

**Fig. 11:**
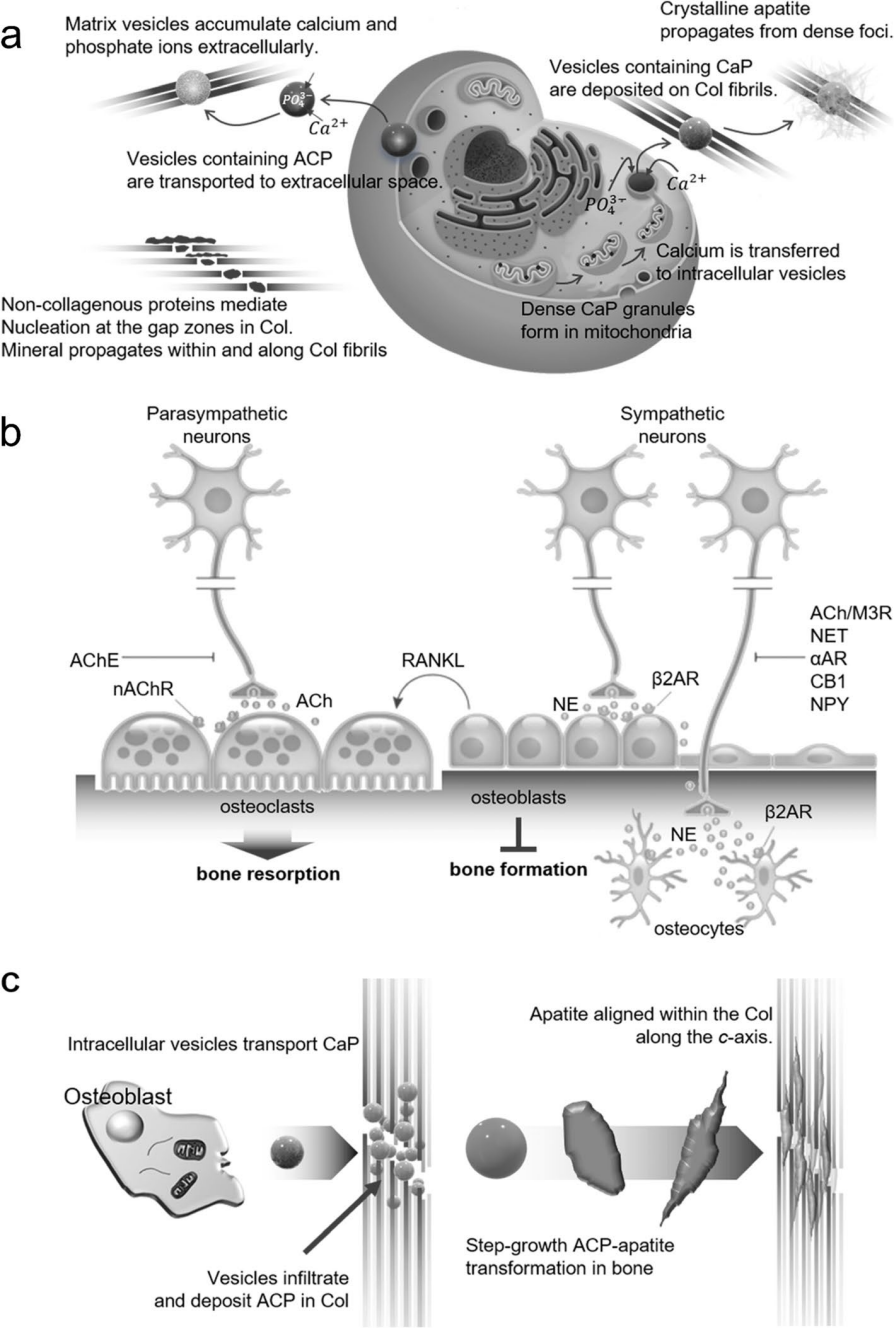
**a** Representation of model and mechanism for bone mineral formation [147]. **b** Representation diagram of mechanism by sympathetic nerves affecting bone formation inhibition and resorption promotion [148]. **c** Diagram describing a possible bone mineralization mechanism [149]. Col, collagen; AChE, acetylcholinesterase; nAChR, nicotinic acetylcholine receptor; Ach, acetylcholine; NE, norepinephrine; AR, adrenergic receptor; M3R, muscarinic 3 receptor; NET, norepinephrine transporter; CB, cannabinoid; NPY, anxiolytic neurotransmitter and cotransmitter

Figure 11a shows a suggested model for biomineralization, including mitochondrial granules, vesicles containing calcium and phosphorus and extracellular mineral precipitation [147]. Considering the previous observations of minerals, collagen-based inorganic accumulation in extracellular vesicles, observations of intracellular CaP, and migration into the ECM are schematically shown. The hypothesis is that (i) matrix vesicles accumulate calcium and phosphate ions extracellularly before budding from the cell membrane and are associated with the collagenous ECM [147]; (ii) non-collagenous proteins associated with the collagen cleft region mediate mineral nucleation and promote collagen fibrils and their transmission [118]; and (iii) ACP and calcium ions stored in mitochondria are transported to the ECM via vesicles and converted to crystalline apatite and proliferate in dense foci. Other studies have induced the mechanism by relating bone tissue and nerve cell signals (Fig. 11b) [148]. The noradrenergic nerve terminal in the bone releases norepinephrine and stimulates β2-adrenergic receptors (ARs) near osteoblasts and osteocytes. This acts as a barrier to bone formation and leads to increased expression of the receptor activator of nuclear factor kappa-β ligand (RANKL), which also increases bone resorption due to osteoclast formation. This disrupts bone formation and promotes bone resorption through adrenergic agonist induced bone loss. The initial crystallization steps were studied during the transformation of ACP to HAp and/or other apatite groups using biomimetics and nanocomposites (Fig. 11c) [149]. Starting from (i), intracellular matrix vesicles from osteoblasts transport ACP precursors that are irregular and/or spherical granules into the gap zone of the collagen matrix. (ii) ACP-containing vesicles penetrated the collagen matrix and deposited ACP granules in the gap region. (iii) The deformation of ACP granules into bone apatite groups along the long axis of collagen via a step-flow cluster/lysis-growth mechanism. (iv) Fully transformed and mature mineralized collagen matrix.

It has been clear for many years that hard tissue mineralization occurs in a structured matrix of Col-I, and that the crystalline phase is composed of individual plate-like nanocrystal agglomerates of HAp oriented and positioned by the collagen fibril structure. The mineralization process itself can be considered from several different perspectives [82], which are (1) crystallization in a mineral phase from a supersaturated solution; (2) the presence of protein polyions in the mineralization system and how to interact with nanoclusters of free or calcium and phosphate ions to control the mineralization process; (3) the non-collagenous proteins of hard tissue and methods that can be delivered in vivo to modulate mineralization at specific sites, including the processes of nucleation, crystal growth, morphology, and size control; and (4) delivery of sequestered vesicular nanoclusters of calcium and phosphate directly from the cell or mitochondria to the mineralization front. Enormous amounts of biominerals are produced from carbonates and silicates in plant and animal systems, and in all cases, common mechanisms can be applied.

For research and applications, mammalian skin and tendon tissue are the main sources of Col-I. It should be noted that most previous studies on collagen have been performed using rat tail tendon because of their high purity and easier extraction process than other sources [10]. Besides all the developments in extraction and purification processes, collagen is an animal extract that causes problems with immunogenicity and interspecies infection [150, 151]. The triple helical domains extracted from bovine and porcine collagen are similar to human collagen, but immunologically relevant differences in the telopeptide site can cause an immune response [152]. Peptide digestion cleaves non-helical termini, but their immunogenic potential is not eliminated. Interest in heterologous biological materials lies in the transfer of infectious agents. These concerns have stimulated research into collagen-like synthetic peptides with the cellular production of collagen, human recombinant collagen, and cultural concerns [10].

In recent years, biomineralization of fibrillar collagen directing agents on these collagen-based substrates has allowed scientists to gain insights into their potential mechanisms. A new model for collagen endothelial fibrosis was developed to establish the Gibbs-Donnan equilibrium in a polymer electrolyte directional mineralization system, which complements the existing collagen mineralization mechanism by simultaneously balancing neutrality and osmotic equilibrium.

Charged macromolecules mimicking acidic non-collagenous proteins [153] in bone or dentin, such as pAsp [118], pAA [154], fetuin [119], and polyallylamine hydrochloride (pAH) [132], are essential for in vitro biomineralization of Col-I fibrin. The in vitro biomimetic model has been proven to provide profound insights into collagen mineralization, and various studies have been conducted using this mechanism. As mentioned previously, to ensure the perception of the mechanism of intrafibrillar mineralization, Sone et al. looked at the effect of pAsp molecular weight on the remodeling of mineralization and characterized the mineralization solution [131]. In their study, pAsp inhibited crystallization in solution to slow ACP growth by stabilizing this step rather than isolating Ca^2+^ ions. Ultimately, these results suggest that the Asp-rich protein sequence is essential for the crystallization step and may also be useful for optimizing of synthetic mineralized tissue replacement.

### Advances in synthesis and analysis

#### Synthesis of calcium phosphates under biological condition (Biomimicry)

Synthesis of CaPs under conditions similar to the body is proceeding more actively than under the above-mentioned laboratory conditions. The minerals in bone tissue are similar to HAp, but contain many other ions, as summarized in Table 6. Each element mentioned in Table 6 plays a pivotal role in apatite biological behavior for better biomass production [155]. Studies on the mechanism of nucleation and crystal growth of bone tissue have been conducted with many of these ions as variables. Magnesium has important implications for calcification and bone weakness and indirectly affects mineral metabolism [156]. Although not part of the main fate of human hard tissue, Sr is considered a bone-seeking element that has a beneficial effect on bone growth and can reduce bone absorption and improve bone growth [157]. Zinc is known to promote the proliferation and differentiation of osteoblasts and is widely used in the fabrication of biomaterials for bone tissue engineering [158]. Potassium has a multipurpose nature in the control of biochemical processes and plays an important role in the nucleation of apatite minerals [159]. Sodium is known to play a potential role in cell adhesion, bone metabolism, and bone resorption and may also be added as a bioactive glass component [31]. Chlorine can assist the acidic environment on the bone surface to activate osteoclasts in the bone resorption stage [160].

Synthesis techniques for the nucleation and growth of apatite using solutions, such as SBF, similar to the chemical environment of the blood present in the human body, have been used. Recently, studies on bone growth and bone regeneration mechanisms have been conducted with SBF or m-SBF; however, they have mainly been applied for biomineralization or bioactivity verification of developed materials or applications such as medical device surface treatment [161, 162]. Thus, the surfaces of metal implants modified with inorganics have been developed very quickly and are known to be very helpful for patient treatment. However, no implant has uniformly formed a layer between the implant and real soft or hard tissue. It is not yet clear how the apatite phase undergoes nucleation and crystallization in SBF. To accurately understand the mechanism of bone growth, many scientists have challenged the synthesis of apatite in conditions similar to those in the human body (i.e., temperature, pH, chemical composition, and fluid velocity); however, it is difficult to conclude that the morphology and stage of growth coincide with the real bone [140, 163]. In synthesizing HAp using SBF, selecting raw materials irrelevant to the human body and obtaining amorphous precursors is also a problem. The precursors have to be proved by obtaining HAp or other CaPs through high-temperature heat treatment (calcination), which makes it impossible to confirm the mechanism of the actual growth direction control of HAp crystals [140]. Based on thermodynamic theory, serum and SBF are supersaturated with apatite crystals [164]. The initial state of this system can be referred to as a metastable state, which relies on the non-classical crystallization theory, but by forming HAp crystals, it becomes thermodynamically stable [165]. Eventually, when the HAp is formed from a cluster immersed in serum or SBF, it can be interpreted that the immersion time was longer than the induction time [163]. According to the initial definition of SBF, bioactive materials are substances that accelerate heterogeneous apatite crystallization in solutions supersaturated with HAp [33, 135]. It can be said that to obtain specific energy to induce apatite nucleation and crystal growth, there must be a substrate in the solution rather than a simple precipitation concept. In recent years, synthesis and analytical technologies at the nanometer scale have been developed, and the nucleation and growth mechanisms have been interpreted from various perspectives. Research is also underway on organisms that regulate the size and growth direction of HAp crystals and the spacing between HAp crystals in the bone tissue.

#### Analysis of synthesized materials and bone tissue

Bones are categorized by the relative amount of solid material, size, and number of spaces. There are two types of bones: cortical (compact) and cancellous (spongy). Among all bones, dense bones are thinly located at the surface, and the deep bony bone and medullary cavity are in the sponge bone area. Blood cells and platelets are produced in the medullary cavity between spicules in the adult medullary cavity. Yellow and red bone marrow (where blood cells and platelets are generated) are observed between the spicules of the adult medullary cavity.

The specific structure and ratio of compact to spongy bones differ greatly from their functions. The dense bone provides a force to support the weight. Long bones are designed with attachment and firmness of muscles and ligaments, and the amount of compact bone is highest at the center of the shaft. In addition, the long bone acts as a support to attach a large muscle to the raised part (e.g., crest tubercle). Living bones exhibit elasticity, flexibility, and strong rigidity (or hardness). The various and excellent mechanical properties of bone tissue are due to organic and inorganic materials. From another perspective, in bone, stiffness (290 GPa) coexists with toughness (22 MPa m^−1/2^) under the bone’s hierarchical organization [166]. The basic structure of bone is a hybrid of organic and inorganic materials based on hydrated mineralized Col-I fibrils [167].

The hierarchical assembly of organics and inorganics in the bone tissue is implemented bottom-up through interactions between cells and the ECM during growth, development, and maintenance [19]. However, such a hierarchical bone structure has been approached substantially top-down manner [168]. As expected, this approach can be attributed to the scale of the analytical methodology. Thus, with the development of analytical techniques, it is possible to verify up to the atomic level; simultaneously, it shows an excellent ability to analyze the crystalline direction and shape. The radical development of this analytical technique has led to surprising results for bone structure, allowing researchers to study bone growth mechanisms more objectively. Although the mechanisms of organics (collagen cross-linking into a continuous framework) and inorganics (crystallite aggregation) are different, the results of these studies converge to provide continuity of organic and inorganic components of bone tissue. Bone morphology has been previously demonstrated by deproteinization or demineralization in the hydrated state on several scales [169].

**Fig. 12:**
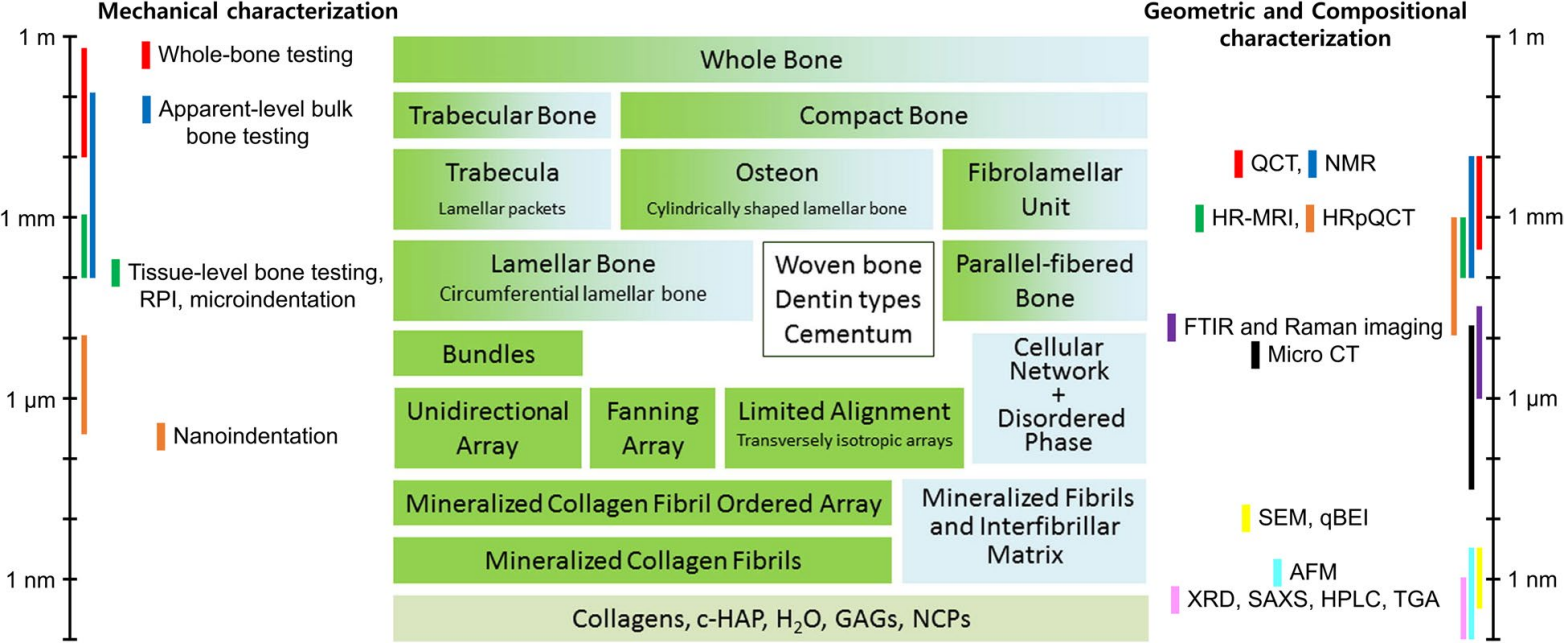
Schematic diagram of the hierarchical organization of bone (center; ordered material, green; disordered material, blue) [168]. Representative techniques for the assessment of the organization of bone tissue (both sides) [170]. GAG, glycosaminoglycan; QCT, quantitative computed tomography; NMR, nuclear magnetic resonance; HR-MRI, high resolution magnetic resonance imaging; HRpQCT, high-resolution peripheral quantitative computed tomography; FTIR, Fourier-transform infrared spectroscopy; CT, computed tomography; qBEI, quantitative backscattered electron image; XRD, X-ray diffraction; SAXS, small-angle X-ray scattering; HPLC, high performance liquid chromatography; TGA, thermal gravimetric analysis

It is no exaggeration to mention that the development of analytical technology has played a major role in the analysis of hard tissue according to the scale (Fig. 12). Hard tissue analysis can be confirmed by the development of analytical techniques, as well as by the chemical elements, structure, and patterns that constitute the hard tissue. However, it is not a simple task to combine the results obtained with existing methods because the existing analysis methods are not based on the actual requirements of the living organism. In recent years, research on hard tissue has been actively conducted, as the technology for analyzing the environment as an actual living organism has been confirmed by the modification and improvement of existing methods.

Mechanical events in biological systems are a major problem in bone tissues [171]. In general, whole-bone testing involves stabilizing, loading, and measuring the resulting deformation of the bone of interest. The disadvantage is that it is intrinsically destructive, and analysis at this level can separate structural elements that would affect structural properties. Bulk compact and sponge bone tissues can be prepared in standard-sized specimens and subjected to mechanical testing to determine the properties of the biomaterials for each tissue, including apparent stiffness, strength, and toughness. While these tests have proven useful in explaining the effects of drug therapy, disease, and aging [172–174], the drawback of evaluating mechanical properties at this level is the difficulty of uniformly processed specimen preparation procedures. Typical dimensions for tissue-level mechanical testing are hundreds to thousands of microns, and owing to the technical problems of testing such small specimens, the microtensile or microbeam bending test is preferred over the micro-compression test. In addition, the estimation of the properties of mechanical test specimens at the tissue level from the structural properties of the whole bone is cumbersome and requires several basic assumptions, including material homogeneity and prismatic cross-section [175]. Indentation (micro-indentation, RPI; reference point indentation, nano-indentation) tests can be performed on multiple length scales to provide information on the resistance of bone tissue to plasticity and permanent deformation. Tissue hydration, tip shape, surface roughness, loading rate, and specimen orientation must be considered in the indentation tests [176].

The properties of HAp-like minerals, Col-I, and body fluids in bone tissue provide insights into the quality of bone and contribute to the structural integrity of the whole bone [177]. Nuclear magnetic resonance enables determining the water content in bone tissue to determine the compact and trabecular bone mineral density [178, 179]. This method allows the body to be scanned without the organism being exposed to ionizing radiation and can be repeated on a nondestructive, identical patient or sample. However, because of the low signal-to-noise ratio (SNR) of isotopes other than ^1^H, the problem is that they are limited to samples with high water content, such as biological tissues [180]. The molecular bond vibration properties of materials-dependent infrared and Raman spectroscopy confirmed the chemical properties of the sample. These methods can be used to distinguish the molecular signals of the organic matrix component from the signal generated from the HAp component. The advantage of using SEM, which can be rasterized across sample surfaces with an electron beam to determine topographical and compositional information at resolutions of tens of nanometers, is that the optical microscope eliminates the need for higher resolution and thinner sections than standard histology [181]. In addition to this common SEM, SEM imaging produces backscattered electron signals that are used to generate qBEI. The number of backscattered electrons is proportional to the atomic number Z of the atom with which it collides, also known as the Z contrast. Among the bone constituents, the Z number of calcium was the highest, and calcium dominated the qBEI image contrast and showed different stages of mineralization in the tissue. As a result, qBEI is well-suited for bone mineral density distribution measurements that spatially detail the degree of mineralization across the region of interest [182]. Thermal gravimetric analysis monitors the weight change of a sample as it changes the temperature to characterize the mass of organic and inorganic components in the sample. TGA is the gold standard for determining the fraction of minerals in bone tissue but has the disadvantage of having a destructive step that leaves only minerals after combustion.

The composition and distribution of compact and trabecular bones vary with the entire skeleton and bone function. Choosing the correct scale is important for selecting techniques to characterize their geometry. Magnetic resonance imaging (MRI) is a widely used non-ionized clinical form used to image water in skeletal tissue in the body. Specific propagation by MRI has a huge advantage in images related to the tissue type (bone, fat, and marrow). This method can efficiently identify the structural information of the hard tissue to be measured based on the structural dependence of the local field induced near the bone and bone marrow boundaries [183]. However, there are drawbacks to the low SNR and high cost of trabecular bone analysis, which continue to be studied [183]. Quantitative computed tomography can measure 3D bone structure and volumetric bone mineral density (vBMD) in the body. This analysis can distinguish between the compact and trabecular compartments; however, it is insufficient to characterize the microstructure of the individual struts of the trabecular bone.

In contrast, HRpQCT has the advantage of enabling in vivo measurement of vBMD and resolution of compact and trabecular bone features. This analysis has excellent resolution and is used in studies that show microstructural differences such as osteoporosis [184]. Micro-computed tomography has been used to characterize the microarchitecture of trabecular bone and the 3D bone structure of biopsies, other excised tissues, or small animal skeletal areas in the body. In small animals, this analyzer can monitor skeletal morphology over time, and the human biopsy process can determine the impact of disease states on bone morphology [185, 186]. Solving the problem of long scan times and radiation exposure for high resolution is a practical analysis method. AFM measures the deformation of forces that occur when a cantilever beam with a sharp tip on an atomic surface is scanned at the surface of the specimen, resulting in terrain imagery at a subnanometer spatial resolution [187]. The analysis also has excellent applicability for manipulating sample surfaces and measuring force. This technology can be imaged and probed in saline or other liquids compared to other imaging techniques, making it a key advantage in analyzing near-biological conditions. For ex vivo studies, XRD is a gold standard that characterizes bone mineral structures. A century ago, HAp was the first XRD analysis of bone-established minerals. Since then, XRD analysis technology has steadily evolved, leading to significant insights into crystallinity and mineralization as a function of tissue and animal age, the effects of crystallization on mineral deformation, and impurity substitution [188]. Small-angle X-ray scattering is a complementary technology to XRD that does not require sample homogenization. This analysis provides information on the crystal shape, thickness, and orientation [189]. The main advantage of this method is that it allows imaging of amorphous specimens with minimal specimen preparation, thus providing detailed insight into the structure of bone tissue at the nanoscale, changes in tissue mineral density, and damage accumulation.

#### Biomimetic materials for bone regeneration (Overview) – limitation of current methods

Research on bone growth and regeneration has been ongoing for decades, and materials used in clinical trials have also been steadily developed. In the early stages of research on bone growth and regeneration, research was conducted in basic science on the chemical composition and structure of natural bones, focusing on the development of metallic materials with a focus on mechanical properties. Since then, research and development of CaP-based materials constituting natural bone has been developed in the field of engineering, and numerous bone grafts have been developed for clinical applications. Since the 1990s, as interest in polymer materials has increased, new composite materials have been developed to compensate for the disadvantages of CaP-based materials and other ceramic materials. Research on collagen started doing so, became interested in various major fields and became alive again. The development of the analytical technology mentioned above has also contributed to the research and development of materials using chemicals identical to natural bones that became active over time. This section describes biomimetic materials for bone regeneration, which are of recent interest, as well as new synthetic processes, fusion analysis techniques, and limitations.

Shao et al*.* used CaP ion clusters to induce epitaxial crystal growth of tooth enamel apatite in hard tissue [190]. The material obtained was proven to have the same hierarchical structure and mechanical properties as natural enamel. A promising strategy for the biomimetic regeneration of substances with complex structures, such as bone tissue, with epitaxial growth based on CaP phase transformation, is also presented. Another study showed evidence based on the structural role of water concerning the direction and shape of crystal growth. In general, it is known that organic molecules from the vertebrate ECM of calcified tissues are essential for structuring apatite minerals. Wang et al*.* demonstrated the structure and bone tissue of crystalline and biomimetic apatite nanoparticles according to temperature variables using various analytical techniques [191]. Their results showed that water-oriented apatite crystals formed through an amorphous CaP-like layer coating the crystalline core of the bone apatite. This disordered layer is reminiscent of what was found around the crystal nuclei of calcified biominerals through various parameters in vivo and provides an extended local model of biomineralization.

In practice, it is still difficult to identify the differences between natural bones and changes in material implanted at the site of defects during bone tissue regeneration. To solve these problems, Li et al*.* conducted a study that compared HAp doped with terbium (Tb) as a bone graft material with the surrounding natural bone apatite [192]. This study demonstrated the occurrence and gradual decomposition of compositional and structural changes in implanted materials during bone regeneration and reconstruction, thereby revealing significant differences from bone apatite crystals. Synthetic nano-HAp crystals with high crystallinity implanted in the bone defect site could be bone-fused with the bone tissue; however, they were slowly degraded by being treated as foreign matter at the implanted tissue site. These results suggest that biomimetic bone-tissue regeneration materials can be developed and applied. From another point of view, it is an important factor in determining the results of bone tissue regeneration, and it is also applied based on the theory of the host immune response to bone biomaterials. In this regard, Jin et al*.* conducted a bone regeneration study using immunomodulatory properties and mesenchymal stem cell recruitment techniques during endogenous bone regeneration [193]. This study induced new bone formation using acquired hierarchical intra-fibrillary mineralized collagen through CD68^+^CD163^+^ M2 (cluster of differentiation, M2; alternatively activated macrophages) macrophage polarization and CD146^+^STRO-1^+^ (STRO-1, a gene for a protein marker of mesenchymal stem cells) host mesenchymal stem cell recruitment, and has also been shown to promote endogenous bone regeneration by promoting interleukin secretion. This is not just proof of biological factors, but it is also very interesting that it is based on a mineralized collagen matrix in a biomimetic hierarchical fiber with a hierarchical nano-interface.

In tissue engineering, continuous research and development are focused on mimicking natural bone tissue by integrating the structures and functions of the scaffolds. In terms of the complexity of the hierarchical structure, the requirements for mechanical properties, and the diversity of cells residing in the bone tissue, it remains a major challenge to build a biomimetic bone tissue engineering scaffold. Various structures have been developed based on 3D printing technology, which has been steadily concentrated for approximately 10 years. Based on these technologies, Zhang et al*.* developed a scaffold with a hierarchical Haversian bone structure that can act as a capillary tube in the bone tissue [194]. Scaffolds with these Haversian bone mimic structures induced bone tissue formation, angiogenesis, and neurogenic differentiation in vitro. This provides a new strategy for designing structured and functional biomaterials that mimic the natural complex bone tissue during bone tissue regeneration. However, additional experimental analyzes to confirm the more objective and original mechanism in the results of these tissue engineering studies could have served as a more useful technology carrier. On the mechanical side, CaP-based materials have the disadvantages of typical brittle ceramic materials. To compensate for these shortcomings, Yu et al*.* manufactured scaffolds using the 3D stacking technology [195]. Previously, a method for controlling the mechanical properties of a scaffold through various materials and geometric motifs has been studied, but recently, the theme of biomimetics has emerged to avoid other artificial materials. To improve the mechanical properties of the scaffold, they applied a rotated plywood structure of bone tissue to 3D printing scaffold manufacturing. The biomimetic rotated layered plywood motif presented in this study demonstrates that it can improve mechanical performance, suggesting that it can reduce fracture propagation, similar to bone tissue. However, these technologies are close to the morphological viewpoint of macro-scales and are composed of materials that do not exist in the human body in terms of chemistry, so it is not enough to express them as biomimetics when viewed from the microscopic side.

Owing to their excellent biocompatibility and biodegradability, studies related to biomimetic composites in tissue engineering have been actively conducted in recent year. Collagen and HAp are the most abundant proteins and main constituents of vertebrate bone tissue. Based on this knowledge, Yu et al*.* developed a lamellar microstructure and incorporated Fe and Mn ions, essential trace elements in the human body, into an intrafibrillar mineral scaffold composed of apatite and collagen to improve osteoinductivity [196]. The authors of this report also mentioned that the scaffold developed in this study provides a simple but practical strategy, judged to be a very good method for identifying bone growth and regenerative mechanisms in terms of basics. Quade et al*.* loaded a biomimetic mineralized collagen scaffold with a signaling cocktail from hypoxia-regulated human mesenchymal stem cell secretion [197]. In a humid environment, signaling factors are released by forming a chemotactic gradient, leading to the direct migration of cells into the scaffold. It was also co-cultured with human reproductive venous endothelial cells to show the entire structure of the blood vessels sprouting across interconnected pores. The fusion of bioactive material systems into collagen-apatite-based scaffolds, and the application of oxygen and nutrients to attract cells in the body has shown great potential for clinical translation. To solve the clinical problems of existing scaffolds developed for hard tissue regeneration, the technology to respond to bacterial infection and osteomyelitis is applied. This study developed a drug-free inorganic biomaterial platform to reveal biomimicry [198]. In addition, biocompatibility, osteoconductivity, and biomineralization have been the focus of developing bio-scaffolds for hard tissue regeneration, and research on scaffolds using natural polymers that mimic the collagen fiber matrix of bone tissue is ongoing [199, 200].

## Summary

Successful biomineralization of synthesized materials, achieved in the past decade, has been a significant step toward the authentic biomimetic process of bone-like hierarchical structures. As clearly stated in the previous studies reviewed here, the developed materials closely mimic some of the most significant features found in bone tissue, such as mineral density, particle size, and crystalline orientation. Nevertheless, it is still unclear how biomimetic materials are similar to natural bone structures. For example, further analysis of the chemical properties of inorganics formed in the intrafibrillar structure is required, particularly analysis involving ions around the mineralized site. This can provide more insight into the mineralization mechanism of organic phenomena, as some ions seem to affect the process. Although ions are present in very small amounts in natural bone mineral matter, the relevance of these ions should not be neglected. Conversely, bioinorganic additives in minerals can improve the bone-regeneration potential of biomimetic bone grafts.

These biomaterials need to be analyzed for their physical and chemical properties to confirm their material properties, and biological experiments, such as in vitro and in vivo tests, are evaluated to verify their biological mechanisms. Finally, in the convergence study of bone tissue growth and regeneration, it is important to induce its mechanisms in different fields (materials, biology, medicine, etc.). However, it is now necessary to link the mechanics of one field with theories of another to derive a more objective new mechanism.

## Data Availability

Not applicable.

## Abbreviations

ACC: Amorphous calcium carbonate
Ach: Acetylcholine
AChE: Acetylcholinesterase
ACP: Amorphous calcium phosphate
AFM: Atomic force microscopy
AR: Adrenergic receptor
Bigi-SBF: HEPES–NaOH buffered SBF (Bigi is the author’s name.)
BMU: Basic multicellular unit
BSP: Bone sialoprotein
CaC: Calcium carbonate
CaP: Calcium phosphate
CaSi: Calcium silicate
CB: Cannabinoid
CDHA: Calcium-deficient hydroxyapatite
cHAp: Carbonated hydroxyapatite
Col-I: Type I collagen
cryo: Cryogenic
c-SBF: Corrected simulated body fluid
DCPA: Dicalcium phosphate anhydrous
DCPD: Dicalcium phosphate dehydrate
DL: Dense liquid
ECM: Extracellular matrix
ECP: Endothelial cell progenitor
ESB: Electron selective backscatter
FAp: Fluorapatite
GF: Growth factor
Glu: Glutamic acid
HAp: Hydroxyapatite
HEPES: N-2-hydroxyethylpiperazine-N-ethanesulfonic acid
HRpQCT: High-resolution peripheral quantitative computed tomography
HSC: Hematopoietic stem cell progenitor of osteoclast
IR: Infrared
i-SBF: Ionized SBF
Leu: Leucine
M3R: Muscarinic 3 receptor
MCPA: Monocalcium phosphate anhydrous
MCPM: Monocalcium phosphate monohydrate
micro CT: Microcomputed tomography
MRI: Magnetic resonance imaging
mRNA: Messenger ribonucleic acid
m-SBF: Modified SBF
MSC: mesenchymal stem cell progenitor of osteoblast
nAChR: nicotinic acetylcholine receptor
NE: norepinephrine
NET: norepinephrine transporter
NMR: nuclear magnetic resonance
NPY: anxiolytic neurotransmitter and cotransmitter
n-SBF: improved SBF
Ob: osteoblast
OC: osteocalcin
Oc: osteoclast
OCP: octacalcium phosphate
ON: osteonectin
OP: osteopontin
pAA: polyacrylic acid
pAH: polyallylamine hydrochloride
pAsp: polyaspartic acid
pH: potential hydrogen
PICP: procollagen type I C propeptide
PINP: procollagen type I N propeptide
PTH: parathyroid hormone
qBEI: quantitative backscattered electron image
QCT: quantitative computed tomography
RANKL: receptor activator of nuclear factor kappa-β ligand
RPI: reference point indentation
r-SBF: revised SBF
SAED: selected area electron diffraction
SAXS: small-angle X-ray scattering
SBF: simulated body fluid
SCI: science citation index
SEM: scanning electron microscopy
Ser: serine
SNR: signal-to-noise
SS: supersaturated solution
SSEEL: Ser-Ser-Glu-Glu-Leu
Tas-SBF: 27 mM HCO3− TRIS–HCl buffered SBF (Tas is the author’s name.)
TCMP: tricalcium phosphate substituted by magnesium
TCP: tricalcium phosphate
TEM: transmission electron microscopy
TGA: thermal gravimetric analysis
vBMD: volumetric bone mineral density
XRD: X-ray diffraction
αAR: alpha-adrenergic receptor
